# Regulation of the Microbiome in Soil Contaminated with Diesel Oil and Gasoline

**DOI:** 10.3390/ijms26136491

**Published:** 2025-07-05

**Authors:** Agata Borowik, Jadwiga Wyszkowska, Magdalena Zaborowska, Jan Kucharski

**Affiliations:** Department of Soil Science and Microbiology, Faculty of Agriculture and Forestry, University of Warmia and Mazury in Olsztyn, 10-719 Olsztyn, Poland; agata.borowik@uwm.edu.pl (A.B.); m.zaborowska@uwm.edu.pl (M.Z.); jan.kucharski@uwm.edu.pl (J.K.)

**Keywords:** rhizosphere, bacteriome, mycobiome, microbial stress, maize

## Abstract

Petroleum-derived contaminants pose a significant threat to the soil microbiome. Therefore, it is essential to explore materials and techniques that can restore homeostasis in disturbed environments. The aim of the study was to assess the response of the soil microbiome to contamination with diesel oil (DO) and gasoline (G) and to determine the capacity of sorbents, vermiculite (V), dolomite (D), perlite (P) and agrobasalt (A), to enhance the activity of microorganisms under *Zea mays* cultivation conditions in pot experiments. The restoration and activity of the soil microbiome were evaluated based on the abundance and diversity of bacteria and fungi, using both classical microbiological methods and Next Generation Sequencing (NGS). Bioinformatic tools were employed to calculate the physicochemical properties of proteins. DO increased the abundance of cultured microorganisms, whereas G significantly reduced it. Both DO and G increased the number of ASVs of *Proteobacteria* and decreased the relative abundance of *Gemmatimonadetes*, *Chloroflexi*, *Acidobacteria*, *Verrucomicrobia*, *Planctomycetes*, and fungal OTUs. These contaminants stimulated the growth of bacteria from the genera *Rhodanobacter*, *Sphingomonas*, *Burkholderia*, *Sphingobium*, and *Mycobacterium*, as well as fungi belonging to the *Penicillium* genus. Conversely, they had a negative effect on *Kaistobacter*, *Rhodoplanes*, and *Ralstonia*, as well as the fungi *Chaetomium*, *Pseudaleuria*, and *Mortierella*. DO caused greater changes in microbial alpha diversity than G. The stability of microbial proteins was higher at 17 °C than at −1 °C. The most stable proteins were found in bacteria and fungi identified within the core soil microbiome. These organisms exhibited greater diversity and more compact RNA secondary structures. The application of sorbents to contaminated soil altered the composition of bacterial and fungal communities. All sorbents enhanced the growth of organotrophic bacteria (Org) and fungi (Fun) in DO-contaminated soils, and actinobacteria (Act) and fungi in G-contaminated soils. V and A had the most beneficial effects on cultured microorganisms. In DO-contaminated soils, all sorbents inhibited the growth of *Rhodanobacter*, *Parvibaculum*, *Sphingomonas*, and *Burkholderia*, while stimulating *Salinibacterium* and *Penicillium*. In G-contaminated but otherwise unamended soils, all sorbents negatively affected the growth of *Burkholderia*, *Sphingomonas*, *Kaistobacter*, *Rhodoplanes*, *Pseudonocardia*, and *Ralstonia* and increased the abundance of *Gymnostellatospora*. The results of this study provide a valuable foundation for developing effective strategies to remediate soils contaminated with petroleum-derived compounds.

## 1. Introduction

Many countries are struggling with serious environmental pollution caused by the presence of toxic organic compounds as a result of dynamic expansion and increased industrial activity. Petroleum products are one of the most significant groups of these pollutants. They seep into the soil, causing negative ecological consequences and often leading to environmental disasters and, consequently, contributing to social tensions in affected regions [1]. The BP report [2] predicts that, between 2025 and 2030, the global demand for and consumption of crude oil will fluctuate between 80 and 100 million barrels per day and that global oil production will reach 12,000–14,500 million metric tons in 2025. More significant in the context of this study are reports on fossil fuel emissions generated by increased oil consumption. According to the Global Carbon Project [3], these emissions reached 37.4 billion tonnes of CO_2_ in 2024. It is also estimated that between 1.7 and 8.8 million tonnes of oil are released into the environment annually from natural and anthropogenic sources [4].Therefore, soil contamination with petroleum products such as diesel oil and gasoline is a significant environmental problem [5], affecting not only the physicochemical properties of the soil [6,7], but also altering the structure and activity of the soil microbiome [8,9]. These petroleum products exhibit toxicity to numerous soil organisms, leading to biological imbalances, reduced biodiversity, and impaired biogeochemical processes critical to ecosystem health [10].

Petroleum derivatives, due to their toxicity, mutagenicity, and ability to disrupt biological processes, have a negative impact on the microbial balance of soil, its physicochemical properties, and its ability to maintain essential ecosystem functions. In order to better represent the soil microbiome in biogeochemical models of pollutant degradation, it is worth using genomic data to identify key functional characteristics of microorganisms [11]. In response to such stressors, microorganisms undergo molecular adaptations, including changes to their secondary and tertiary structures. These changes affect the stability of molecules, their ability to perform catalytic and regulatory functions, and the efficiency of translation and transcription [12]. Therefore, studying the structural stability of nucleic acids (RNA and DNA) is an urgent task for molecular biology, as these molecules are highly sensitive to changing environmental conditions, making them difficult to store and use in scientific research. They form complex secondary and tertiary structures, contributing to the control of processes through specific mechanisms, and their prediction is crucial for understanding the functions and regulation of biological processes [13].

In the event of contamination, remediation processes, and particularly phytoremediation, are becoming increasingly important as environmentally friendly methods of soil purification [14,15,16,17,18]. One particularly promising approach is the use of forage plants, which have a low ability to translocate contaminants to their above-ground parts. This minimizes the risk of secondary contamination within the food chain. Examples of such species include *Medicago sativa* (alfalfa), as indicated by Mathur and Panwar [19]; *Lolium perenne* (perennial ryegrass), *Trifolium repens* L., and *Poa pratensis* L., as listed by Dąbrowski et al. [20]; *Festuca arundinacea* (tall fescue), according to Das et al. [21]; and *Zea mays* L. (maize) and *Sorghum bicolor* L. Moench (sorghum), according to Ahmad et al. [22]

Soil remediation using plants involves the absorption, translocation, accumulation, and transformation of pollutants as a result of metabolic activity. Plant roots facilitate the decomposition of pollutants in the rhizosphere by stimulating the activity of archaea, bacteria, and fungi. This is possible because of secretions like sugars, amino acids, phenols, and organic acids, which are a source of carbon and energy for microorganisms [23]. These processes contribute to improved soil quality, fertility, and agricultural suitability. The high potential of phytoremediation, combined with sustainable biocatalytic processes, may offer an alternative method of converting plant biomass into renewable energy sources [24,25].

Although plant-assisted bioremediation methods are gaining in importance, the effectiveness of mineral sorbents in removing petroleum-derived substances is being increasingly verified [16,26,27,28,29]. Due to their layered structure and the presence of exchangeable cations, sorbents can benefit the physicochemical properties of soil, including pH, organic carbon content, and exchangeable cation capacity. These properties can stimulate plant and microorganism growth by absorbing pollutants and creating an environment conducive to detoxification [28,29,30,31,32,33]. Our own research focused on four types of sorbents: agrobasalt, dolomite, vermiculite, and perlite. Agrobasalt and dolomite are rich in nutrients [34]. Vermiculite is a clay mineral composed of magnesium, aluminum, iron, silicon, calcium, potassium, and titanium [30,31,32,33]. Perlite is an amorphous volcanic rock that is rich in silicon, aluminum, potassium, sodium, iron, calcium, and magnesium [26,27,35,36,37,38].

Previous studies on the remediation of soils exposed to petroleum products have focused mainly on changes in their chemical properties and enzymatic activity [26,39]. However, there is a lack of comprehensive analyses integrating the classical microbiological approach with modern sequencing methods and analysis of the molecular structures of microorganisms. Therefore, the aim of this study was to assess the impact of vermiculite, dolomite, perlite, and agrobasalt on the soil microbiome contaminated with diesel oil and gasoline under *Zea mays* cultivation conditions. The study aimed to determine the degree of impact of the sorbents used on the restoration and activity of the soil microbiome, defined through a compilation of classical methods and high-throughput sequencing with analysis of the physicochemical properties of microbial proteins using bioinformatics tools, ensuring an innovative aspect of the research problem in question. As a result, the obtained results may be of significant importance for the development of effective remediation strategies for soils degraded by petroleum compounds.

## 2. Results

### 2.1. Culturable Microorganisms

The study results clearly demonstrated that both the type of soil contamination with petroleum hydrocarbons and the applied sorbents significantly affect the abundance, growth rate, and ecophysiological diversity of the studied microbial groups, which is crucial for the efficiency of soil remediation processes.

**Figure 1:**
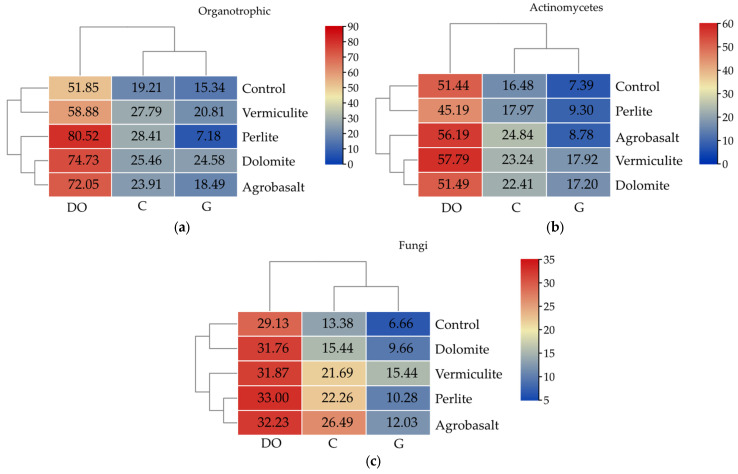
Abundance of culturable microorganisms in 1 kg of uncontaminated soil (C) and soil subjected to diesel oil (DO) or gasoline (G) pressure, depending on the sorbent applied: (**a**)—organotrophic bacteria, 10^9^, (**b**)—actinomycetes, 10^9^, (**c**)—fungi, 10^7^.

The abundance of all studied groups of soil microorganisms was significantly altered by petroleum-derived substances (Figure 1). Soil contamination with diesel oil (DO) increased the abundance of organotrophic bacteria (Org) by 170%, actinomycetes (Act) by 212.14%, and fungi (Fun) by 118%. In contrast, soil contamination with gasoline (G) significantly reduced the abundance of Org by 20%, Act by 55%, and Fun by 50%.

The abundance of microorganisms was also significantly affected by sorbents. The highest abundance of organotrophic bacteria was recorded in uncontaminated soil after the application of perlite, and the lowest abundance was recorded in the control soil, i.e., soil to which no sorbents were added. The abundance of Org increased by 48% with perlite (P), by 45% with vermiculite (V) and by 33% with dolomite (D). Agrobasalt (A) was most beneficial for actinomycetes and fungi, contributing to increases in Act abundance of 51% and Fun abundance of 98%. P was the least effective in promoting the abundance of actinomycetes while D was the least effective in shaping the abundance of fungi. P increased the abundance of Act by only 9%, while D increased the abundance of Fun by 13%.

In soils contaminated with diesel fuel, P reduced the abundance of Act by 12%, whereas V increased it. All sorbents increased the abundance of Org in the range from 14% (V) to 55% (P) and Fun from 9% (D) to 13% (P). The application of perlite to gasoline-contaminated soil, as well as to diesel oil-contaminated soil, resulted in a decrease in the number of Org, but by a much greater extent: as much as 53%. The application of all sorbents (V, D, P, and A) had a positive effect on the multiplication of Act and Fun in gasoline-contaminated soil. As a result, Act abundance increased from 19% (A) to 142% (V), and Fun abundance increased from 45% (D) to 132% (V).

**Figure 2:**
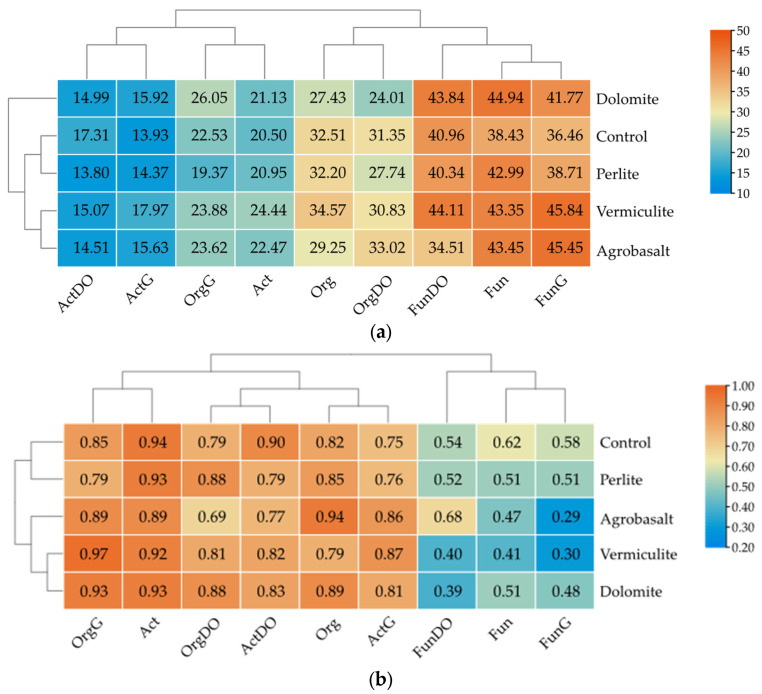
The effect of sorbents and diesel oil (DO) and gasoline (G) on indicators of microbial development in soil: (**a**) colony development (CD), (**b**) ecophysiological diversity (EP). Org—organotrophic bacteria; Act—actinomycetes; Fun—fungi; DO—soil contaminated with diesel oil; G—soil contaminated with gasoline.

The colony development index (CD) of individual groups of microorganisms varied depending on the type of soil contaminated with petroleum-derived substances and the sorbent used (Figure 2a). In soil C, Fun grew the fastest (38.43) and Act the slowest (20.50). DO contamination decreased the growth rates of Org (31.35) and Act (17.31), while increasing that of Fun (40.96). G caused greater changes in the succession of microorganisms than DO, as the CD indices varied to a greater extent. The CD values were 22.53 for Org, 13.93 for Act and 36.46 for Fun under the influence of this contamination.

The sorbents also modified the rate at which microorganisms multiplied. In soil C, sorbent V increased the number of fast-growing Org at the expense of slow-growing ones, while D and A decreased it, and P had no effect. Vermiculite had the strongest effect on the development of Act (24.44). D and A did so as well, but to a lesser extent, while P did not change their growth rate. All sorbents significantly affected the growth dynamics of Fun. Under their influence, the CD value increased from 38.43 (C) to 42.99 (P) and 44.94 (D). Sorbents V and A had an indirect effect. In soil contaminated with DO, sorbents D and P significantly reduced the rate at which Org multiplied. The CD values were 24.01 and 27.74, respectively. Sorbent A, on the other hand, accelerated it (33.02). All sorbents significantly reduced the CD of actinomycetes, while sorbent A also decreased the CD of fungi. The effect of V and D on the CD of fungi was opposite to that of A.

In soil contaminated with G, sorbents D and P had the greatest effect on Org. The first sorbent increased CD from 22.53 to 26.05, while the second reduced it to 19.37. All sorbents increased CD Act and Fun. V had the greatest effect, while P had the least.

Act showed the highest ecophysiological diversity index (EP) value (0.94) in soil C, while Fun showed the lowest (0.62). The introduction of various sorbents into the soil had a varied effect on EP values. Sorbent A increased EP from 0.82 (C) to 0.94 and decreased EP Act from 0.94 (C) to 89. All sorbents decreased EP Fun. V had the greatest effect. Under the influence of this sorbent, EP decreased from 0.62 (C) to 0.41.

Soil contamination with petroleum-derived substances reduced the ecophysiological diversity indices for most groups of microorganisms. Analysis of the ecophysiological diversity (EP) indices of microorganisms in soils contaminated with DO and G allowed us to assess the ability of microorganisms to adapt to changing environmental conditions (Figure 2b). The results obtained prove that DO contamination reduces EP. The EP values for Org were highest in soil with D and P (0.88) and lowest in soil with D (0.69). All sorbents reduced the EP of actinomycetes. The EP fluctuations of these microorganisms ranged from 0.82 (V) to 0.77 (A). Its value in soil without sorbents, however, was 0.90. Fungi, on the other hand, showed the highest diversity in soil with A (0.68) and the lowest in soil with D (0.39) and V (0.40).

In soil contaminated with G, the highest EP value for Org was formed by sorbent V (0.97). The highest EP Act was also in soil with V (0.87) and additionally with A (0.86), while in the case of fungi, the lowest EP was recorded in soil with A (0.29) and V (0.30).

### 2.2. The Abundance of Non-Cultivable Microorganisms

Petroleum-derived contaminants cause significant alterations in the structure of the soil bacterial microbiome, affecting the balance among dominant bacterial phyla such as Proteobacteria, Acidobacteria, and Firmicutes. The application of mineral sorbents modifies these changes by selectively promoting or inhibiting the development of specific tax-onomic groups. The results suggest that the selection of appropriate sorbents, such as vermiculite (V), may serve as an effective tool for targeted shaping of the bacterial micro-biome in soils contaminated with petroleum hydrocarbons (Figure S1). Both products increased the number of ASVs affiliated with the phylum Proteobacteria, and G additionally increased the abundance of the phyla Firmicutes and Bacteroidetes. DO and G both reduced the abundance of bacteria belonging to the phyla Gemmatimonadetes, Chloroflexi, Acidobacteria, Verrucomicrobia, and Planctomycetes. The most abundant phyla in each soil sample were bacteria belonging to the types Proteobacteria and Actinobacteria, as well as Acidobacteria and Firmicutes. The application of mineral sorbents to uncontaminated soil negatively affected the proliferation of Proteobacteria, Actinobacteria, Acidobacteria, Verrucomicrobia, and Planctomycetes. In DO-contaminated soil, all sorbents reduced the number of Proteobacteria, while sorbents D and V increased the number of Actinobacteria, and sorbents A and P reduced it. Sorbents A, P, and V had a positive effect on the Firmicutes phylum, while sorbent G had a negative effect. Sorbents P and V stimulated the abundance of Acidobacteria, while sorbent D inhibited it. In soil samples contaminated with G, all sorbents reduced the abundance of ASV Proteobacteria, Acidobacteria, Verrucomicrobia, and Planctomycetes. Sorbent A had the most adverse effect on Proteobacteria, while sorbents A, P, and V had the most adverse effect on Verrucomicrobia and Planctomycetes. There was a significant increase in Firmicutes under the influence of sorbents A, P, and V, and a significant decrease under the influence of sorbent D. All sorbents increased the number of ASV affiliated with Actinobacteria. In conclusion, three clusters can be identified regarding the impact of the studied variables. The first cluster is a response to the action of DO together with sorbents; the second cluster corresponds to the response to G together with sorbents; and the third cluster is a response to the action of sorbents on bacteria in uncontaminated soil.

**Figure 3:**
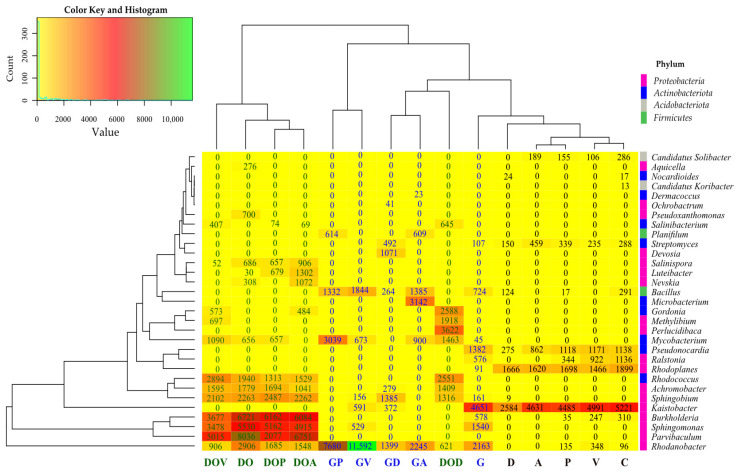
Effect of sorbents and petroleum-derived products on the abundance (ASV ≥ 1%) of bacterial genera in soil. C—uncontaminated soil; V—uncontaminated soil with vermiculite; D—uncontaminated soil with dolomite; P—uncontaminated soil with perlite; A—uncontaminated soil with agrobasalt; G—soil contaminated with gasoline; GV—gasoline-contaminated soil with vermiculite; GD—gasoline-contaminated soil with dolomite; GP—gasoline-contaminated soil with perlite; GA—gasoline-contaminated soil with agrobasalt; DO—soil contaminated with diesel oil; DOV—diesel oil-contaminated soil with vermiculite; DOD—diesel oil-contaminated soil with dolomite; DOP—diesel oil-contaminated soil with perlite; DOA—diesel oil-contaminated soil with agrobasalt.

Analysis of the microbiological composition of soil samples also revealed significant differences in bacterial taxa (Figure 3). In the control soil (C), the most abundant genera from the phylum Proteobacteria were *Kaistobacter* (5221 ASV), *Rhodoplanes* (1899 ASV), *Ralstonia* (1136 ASV), and *Burkholderia* (310 ASV). From the *Actinobacteriota* phylum, the most abundant genera were *Pseudonocardia* (1138 ASV) and *Streptomyces* (288 ASV). From the Firmicutes phylum, the most abundant genus was *Bacillus* (291 ASV). From the Acidiobacteriota phylum, the most abundant genus was *Candidatus Solibacter* (286 ASV). All of the sorbents negatively affected the growth of the following bacterial genera: *Kaistobacter*, *Rhodoplanes*, *Ralstonia*, *Pseudonocardia*, *Bacillus*, and *Candidatus Solibacter*. In contrast, sorbents A and P stimulated the growth of bacteria from the genus *Streptomyces*, whereas sorbents D and V inhibited it.

In soil contaminated with G and DO, bacteria from the genera *Rhodanobacter*, *Sphingomonas*, *Burkhordelia*, *Sphingobium*, and *Mycobacterium* were present. Additionally, G stimulated the growth of *Bacillus* bacteria, which DO did not. Unlike G, DO enhanced the growth of *Rhodococcus*, *Achromobacter*, *Pseudoxanthomonas*, *Aquicella*, and *Salinispora* bacteria. Both DO and G soil contamination had a negative effect on bacteria of the genera *Kaistobacter*, *Rhodoplanes*, and *Ralstonia*.

The impact of sorbents in soil contaminated with DO and G was not unambiguous. In soil contaminated with DO, all sorbents inhibited the growth of bacteria from the genera *Rhodanobacter*, *Parvibaculum*, *Sphingomonas*, and *Burkholderia*, while stimulated the growth of *Salinibacterium*. Sorbents V and D also had a positive effect on bacteria of the genera *Rhodococcus*, *Methylibium*, and *Gordonia*, sorbents V, P, and D on bacteria of the genus *Mycobacterium*, and all sorbents except P on *Salinibacterium*. In contrast, in gasoline-contaminated soil, V, P, and A created better conditions for the proliferation of *Rhodanobacterium*, *Mycobacterium*, and *Bacillus*, with P additionally promoting *Planifilum*, A promoting *Planifilum* and *Microbacterium*, and D promoting *Achromobacter*, *Sphingobium*, *Streptomyces*, and *Devosia*. Supplementing uncontaminated soil G with all sorbents had a negative effect on the development of *Burkholderia*, *Sphingomonas*, *Kaistobacter*, *Rhodoplanes*, *Pseudonocardia*, and *Ralstonia*.

A total of 3115 sequences were obtained from bacteria belonging to the genus. Of these, 1170 were isolated from gasoline-contaminated soil, 825 from diesel-contaminated soil and 1120 from uncontaminated soil. All sequences were deposited in the NCBI GenBank database under the accession numbers PQ531260–PQ532934. The bacteria shown in Figure S2 were grouped into three clusters. Of the ten clones representing the first cluster, four belong to the genus *Sphingomonas*, two to *Sphingobium* and *Parvibaculum*, and one each to *Rhodoplanes* and *Nevskia*. The second cluster consists of 16 clones, of which three belong to the genus *Burkholderia*, two to *Bacillus*, and two to *Achromobacter*. The remaining clones belong to the genera *Pseudoxanthomonas*, *Luteibacter*, *Rhodanobacter*, *Pseudomonas*, *Aquicella*, *Methylibium*, *Ralstonia*, and *Salinispora*. The third cluster was the smallest and comprised two clones from the genus *Streptomyces*, as well as one clone each from the genera *Salinibacterium*, *Arthrobacter*, *Rhodococcus*, *Gordonia*, *Mycobacterium*, *Pseudonocardia*, and *Nocardioides*.

**Figure 4:**
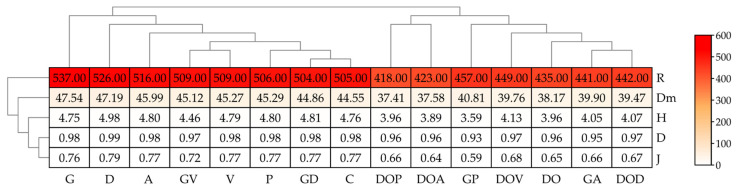
Bacterial diversity indices in soil. H—Shannon; D—Simpson; Dm—Margalef; R—Richness; J—Pielou. C—uncontaminated soil; V—uncontaminated soil with vermiculite; D—uncontaminated soil with dolomite; P—uncontaminated soil with perlite; A—uncontaminated soil with agrobasalt; G—soil contaminated with gasoline; GV—gasoline-contaminated soil with vermiculite; GD—gasoline-contaminated soil with dolomite; GP—gasoline-contaminated soil with perlite; GA—gasoline-contaminated soil with agrobasalt; DO—soil contaminated with diesel oil; DOV—diesel oil-contaminated soil with vermiculite; DOD—diesel oil-contaminated soil with dolomite; DOP—diesel oil-contaminated soil with perlite; DOA—diesel oil-contaminated soil with agrobasalt.

The diversity of bacteria in the soil was determined by both the type of petroleum product and the type of sorbent used (Figure 4). Under the influence of DO, the Shannon index decreased by 17%, Margalef and Richness by 14%, and Pielou by 16%. Only the Simpson index did not change significantly. Gasoline, on the other hand, did not significantly change the Shannon, Simpson, and Pielou indices, but increased the Margalef index by 7% and the Richness index by 6%. In soil uncontaminated with petroleum products, sorbents had a minimal effect on diversity, with only sorbent D increasing the Shannon index by 4%, the Margalef index by 6%, and the Richness index by 4%. The other sorbents did not cause any changes in the values of the analyzed indices. Similarly, in soil contaminated with DO, they did not significantly modify bacterial diversity. However, in soil contaminated with G, sorbent V reduced the Shannon index by 6%, sorbent P by 24%, and sorbent A by 15%. All sorbents reduced the Margalef, Richness, and Pielou indices.

Petroleum-derived contaminants significantly affect the structure of the soil fungal microbiome, reducing its diversity, although some taxa within Ascomycota exhibit adap-tive proliferation. The results indicate a significant role of sorbents, particularly vermicu-lite, in modulating the fungal community structure and supporting microbiome recovery under stress conditions induced by these xenobiotics. Analysis of data on the occurrence of fungi at the phylum level in the soil samples studied indicates a clear dominance of Ascomycota representatives, regardless of the experimental factors used (Figure S3). The number of OTUs ranged from 18,866 (DOA treatment) to 114,322 (GA treatment). The remaining phyla: Basidiomycota, Mortierellomycota, and Mucoromycota occurred in significantly lower abundance. Taking into account the number of OTUs > 1%, the average percentage of each phylum is as follows: Ascomycota 93%, Basidiomycota 2%, Mortierellomycota 4%, and Mucoromycota 1%. Contamination of DO and G soils contributed to a significant reduction in the abundance of all fungal phyla. Supplementation of uncontaminated soil with V contributed to increases in the number of OTUs belonging to Ascomycota (22%), Mortierellomycota (6%), and Mucoromycota (9%), whereas supplementation with P caused increases in OTUs of Basidiomycota (68%) and Mucoromycota (31%). At the other sites, a negative effect of sorbents on fungal abundance was observed. The application of V, D, and P to DO-contaminated soil and V, P, and A to G-contaminated soil stimulated the proliferation of fungi belonging to Ascomycota. The addition of P to soil with DO also increased the abundance of Basidiomycota and Mortierellomycota, while application of A increased Mortierellomycota. In G-contaminated soil, adding D and P increased the number of Basidiomycota OTUs. Conversely, in soil contaminated with G, all sorbents reduced the proliferation of Mortierellomycota and Mucoromycota.

**Figure 5:**
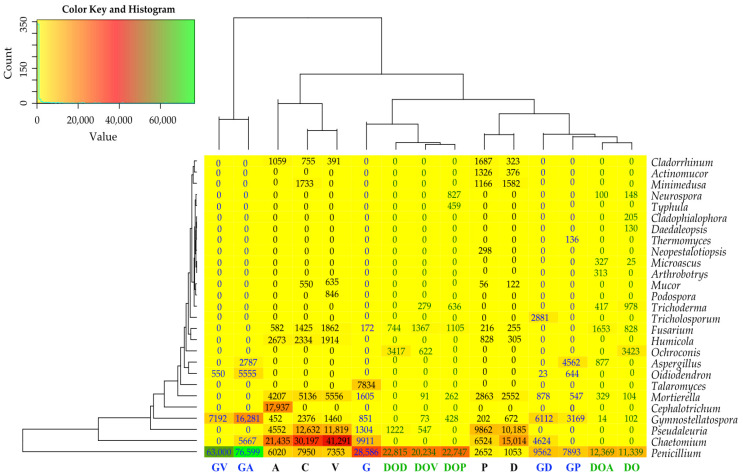
Effect of sorbents and petroleum-derived products on the abundance (OTU ≥ 1%) of fungal genera in soil. C—uncontaminated soil; V—uncontaminated soil with vermiculite; D—uncontaminated soil with dolomite; P—uncontaminated soil with perlite; A—uncontaminated soil with agrobasalt; G—soil contaminated with gasoline; GV—gasoline-contaminated soil with vermiculite; GD—gasoline-contaminated soil with dolomite; GP—gasoline-contaminated soil with perlite; GA—gasoline-contaminated soil with agrobasalt; DO—soil contaminated with diesel oil; DOV—diesel oil-contaminated soil with vermiculite; DOD—diesel oil-contaminated soil with dolomite; DOP—diesel oil-contaminated soil with perlite; DOA—diesel oil-contaminated soil with agrobasalt.

Changes in the composition of the fungal community observed at the phylum level were also evident at the genus level (Figure 5 and Figure S4). Considering the genera of fungi occurring in quantities of OTU > 1%, it was found that uncontaminated soil and soil not supplemented with sorbents were predominantly colonized by representatives of the Ascomycota phylum: the genera *Chaetomium* (46%), *Pseudaleuria* (19%), *Penicillium* (12%), *Gymnostellatospora* (4%), *Humicola* (4%), *Fusarium* (2%), and *Cladorrhinum* (1%). Fungi of the genus *Mortierella* belonging to the phylum Mortierellomycota accounted for 8%, while fungi belonging to the genus *Minimedusa* which is part of the phylum *Basidiomycota*, accounted for 3%. This community structure was disturbed by DO and G soil contamination and the application of sorbents. Petroleum products significantly promoted the proliferation of *Penicillium*, whose OTU number increased from 7950 OTU in the control soil (C) to 11,339 OTU (DO) and 28,586 (G). The abundance of other fungal genera in the soil under the pressure of the analyzed pollutants was significantly lower than in the control sites.

Of the sorbents tested, V was particularly effective, as it created optimal conditions for the growth of *Chaetomium* (an increase of 37% compared to control C), *Fusarium* (an increase of 31%), *Mucor* (an increase of 16%), and *Mortierella* (an increase of 8%) in uncontaminated soil. P and A caused increase in the abundance of *Cladorrhinum* by 124% and 40%, respectively, and A also stimulated the growth of *Humicola* by 15%. The sorbents had a destabilizing effect on other fungal taxa, which was manifested by a lower number of OTUs.

The application of sorbents to contaminated soil led to distinct shifts in the composition of the fungal community. All sorbents in the DO series of experiments caused an increase in the number of OTUs of *Penicillium*, additionally V—*Fusarium*, and P enhanced *Gymnostellatospora*. In turn, in the G series of experiments, all sorbents stimulated the abundance of *Gymnostellatospora*, and additionally V and A—*Penicillium*.

A total of 247 sequences were isolated from uncontaminated soil, 183 from G-contaminated soil and 107 from DO-contaminated soil in the studies conducted. All of these sequences were deposited in the NCBI GenBank database under the accession numbers PQ741026–PQ741430. Figure S4 illustrates the genetic relationships between the fungi identified in the soil, as determined by nucleotide sequences. Three clusters were identified in terms of genetic similarity. The first cluster is the smallest and includes only the *Podila* strain from the phylum Mucoromycota. The second cluster is the largest and consists of seven clones from the genus *Penicillium*, two clones from *Talaromyces* and *Parachaetomium*, and one each from *Chaetomium*, *Cladorrhinum*, *Humicola*, *Gymnostellatospora*, *Fusarium* and *Pseudaleuria*, belonging to the phylum *Ascomycota*; and one *Daedalea* clone representing the Basidiomycota phylum. The third group was the most diverse, and was represented by the genera *Minimedusa* (Basidiomycota) and *Mucor*, *Linnemannia* and *Mortierella* (Mucoromycota).

**Figure 6:**
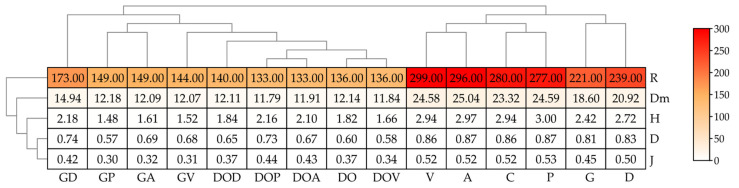
Fungal diversity indices in soil. H—Shannon, D—Simpson, Dm—Margalef, R—Richness, J—Pielou. C—uncontaminated soil, V—uncontaminated soil with vermiculite, D—uncontaminated soil with dolomite, P—uncontaminated soil with perlite, A—uncontaminated soil with agrobasalt, G—soil contaminated with gasoline, GV—soil contaminated with gasoline and vermiculite, GD—soil contaminated with gasoline and dolomite, GP—soil contaminated with gasoline and perlite, GA—soil contaminated with gasoline and agrobasalt, DO—soil contaminated with diesel oil, DOV—soil contaminated with diesel oil and vermiculite, DOD—soil contaminated with diesel oil and dolomite, DOP—soil contaminated with diesel oil and perlite, DOA—soil contaminated with diesel oil and agrobasalt.

The impact of petroleum products and sorbents on changes to the soil mycobiome is evident from the diversity indices (Figure 6). Soil contamination, both DO and G, reduced the value of all coefficients describing the soil mycobiome. The tested sorbents had a heterogeneous effect on the values of fungal diversity index values. The Margalef diversity index (Dm) increased significantly under the influence of V, P and A, while the Simpson (D) and Pielou (J) indices remained unchanged. V and A also caused an increase in the richness index (R). The application of all sorbents to G-contaminated soil resulted in a decrease in all indices describing mycobiome diversity. In the case of soil exposed to DO, all sorbents reduced the Margalef index (Dm). In this series of experiments, the supplementation of soil with D, P, and A, unlike V led to an increase in the Shannon, Simpson, and Pielou indices.

### 2.3. Prediction of Protein Properties Based on Nucleotide Sequences

To better understand the molecular mechanisms underlying microbial adaptation to petroleum-derived contaminants, the predicted physicochemical properties of proteins were analyzed based on nucleotide sequences from isolated bacterial and fungal strains. Bacterial proteins exhibited high glycine and threonine content as well as elevated structural stability. Fungal proteins were characterized by cysteine richness and variable stability, alongside greater sequence diversity in response to the pressure exerted by these contaminants.

An analysis of the predicted physicochemical properties of proteins and their amino acid composition was performed based on nucleotide sequences obtained from isolated bacterial strains derived from soil samples (Table 1 and Table S1). The analysis revealed that glycine (Gly) constituted the highest proportion of amino acids in bacterial proteins, with its content ranging from 32.70% in bacteria isolated from G-contaminated soil to 34.76% in bacteria isolated from C soil. The largest contribution to the protein structure was made by threonine (Thr), with content ranging from 19.11% (object C) to 20.30% (object G). Bacterial proteins were predominantly composed of hydrophobic amino acids. The most stable proteins were found in bacteria identified in the common soil microbiome (G, DO, C). This is evidenced by the instability index value of 38.62. The aliphatic index of the protein was found to have the highest value (average 27.02) in bacteria found in gasoline-contaminated soil. Regardless of where the bacteria were obtained from, the molecular weight of the protein sequences and the overall protein hydrophobicity index (GRAVY) remained similar.

By analyzing the minimum free energy (MFE) of bacteria (Table 2 and Table S2) and the average winter temperature in north-eastern Poland (−1 °C), it can be concluded that bacteria constituting the core bacteriome (G, C), regardless of the type of contamination, have a more stable protein (MFE value = −151.30) than bacteria found in the common soil microbiome. Considering the secondary structure at a temperature of −1 °C, it can be concluded the bacteria that constitute the common microbiome (object G, DO, C) and the microbiome of gasoline-contaminated soils (object G) were characterized by greater (pz) diversity, which was 89.09 and 94.73, respectively. These bacteria had more compact RNA secondary structures. As predicted, higher temperatures reduce protein stability, diversity, and stability of soil microbiome structures.

Cysteine (Cys) was the most abundant amino acid in fungal proteins, accounting for an average of 28.39% of the amino acid composition, across all fungal clones (Table 3 and Table S3). Gly, Ala, and Thr were significantly less abundant, accounting for 24.93%, 23.50%, and 23.12%, respectively. The molecular weight of the peptides ranged from 20.29 kDa (fungi isolated from object C) to 22.75 kDa (fungi isolated from object G), and the instability index ranged from 47.19 (treatment G, DO, and C) to 55.54 (treatment DO). The highest protein aliphatic index values were recorded for fungi constituting the mycobiome of sites G, DO, and G, C. The latter had the highest GRAVY index (0.93), the highest Cys content (31.63%), and the lowest instability index (47.19).

Analyzing the data presented in Table 4, and taking into account the minimum free energy (MFE) of fungi, it can be concluded that fungi have the least stable protein (MFE value of −60.63) in uncontaminated soils. Their diversity in uncontaminated soils was the lowest, and their secondary structure showed less (average MFE value of −54.40). The clones were *Mucor* sp. New. Reference, *Podila* sp. New. Reference, *Linnemannia* sp. New. Reference, *Minimedusa* sp. New. Reference, *Humicola* sp. SH1615601.08, *Fusarium* sp. SH1212089.08, *Cladorrhinum* sp. SH1505060.08, and *Parachaetomium* sp. New. Reference (Table S4). The lowest minimum free energy (average −88.30) was found in the sequences of fungi identified in soils contaminated with DO and G. However, they were characterized by greater population diversity, and their secondary structure did not exhibit high stability. These clones were identified as *Penicillium* sp. New. Reference. As with bacterial proteins, all analyzed fungal sequence proteins were more stable at lower temperatures.

## 3. Discussion

### 3.1. The Influence of Petroleum Products on the Cultivable Microbiome

Environmental contamination with petroleum-derived substances is a widespread issue [6,40]. The presence of these pollutants in the soil leads to its environmental degradation [41,42,43,44]. This is primarily due to the fact that polycyclic aromatic hydrocarbons (PAHs) are highly hydrophobic and tend to adsorb onto organic and mineral particles in the soil [41,42]. The adsorption of petroleum compounds limits the bioavailability of elements for microorganisms, while the toxic components of these substances can also penetrate the cell membranes of microorganisms [43] and plants [44], leading to oxidative stress, DNA damage, and metabolic disturbances. Contamination of soil with diesel oil (DO) and gasoline (G) disrupts the microbiome’s balance, favors microorganisms capable of adapting to chemically stressful conditions. The degradation of petroleum compounds in soils is a complex process influenced by various factors, including soil texture, temperature, pH, and organic matter content. Our study demonstrated significant changes in the abundance of all cultivable microbial groups in the soil. Importantly, diesel oil increased the abundance of organotrophic bacteria (Org), actinomycetes (Act), and fungi (Fun), whereas gasoline significantly reduced it. Gasoline induced more pronounced changes in the microbial succession in soil than diesel oil, which reduced the values of ecophysiological diversity.

The microbial response to petroleum contamination is also explained by the persistence and toxicity of aromatic hydrocarbon compounds. They possess carcinogenic potential, are environmentally persistent, and can cause long-term adverse ecological effects [45,46]. These pollutants therefore raise considerable concern regarding soil fertility and, consequently, food safety [47,48]. The hydrophobic properties of petroleum compounds cause them to interact with the lipid structures of microbial cell membranes [49,50,51,52]. The bacterial cell membrane has a hydrophobic “core,” which serves as a barrier to polar compounds but allows the passive diffusion of lipophilic substances such as hydrocarbons. These compounds dissolve into the lipid bilayer and accumulate within it, thereby disrupting membrane structure and function. Increasing membrane fluidity is essential to mitigate the toxic effects of these substances [52].

### 3.2. The Influence of Petroleum Products on the Non-Cultivable Microbiome

#### 3.2.1. Bacteria

Soil microorganisms respond rapidly to environmental changes, making them sensitive biosensors [53,54]. In our own studies, we observed an increase in the Amplicon Sequence Variant (ASV) of Proteobacteria after the application of both petroleum substances. Additionally, gasoline stimulated the growth of *Firmicutes* and *Bacteroidetes*. Petroleum substances decreased ASV counts of bacteria classified within the phyla Gemmatimonadetes, Chloroflexi, Acidobacteria, Verrucomicrobia, and Planctomycetes. Similar patterns of dominant Proteobacteria and Firmicutes sequences were found in soils contaminated with diesel oil subjected to rhizoremediation [55] as well as in soils near an active oil field [56]. The increase in microbial sequence abundance suggests that these organisms are able to adapt to extreme environmental conditions. Indigenous microbial communities demonstrated strong capabilities for hydrocarbon degradation [55,56,57].

In the study by Galitskaya et al. [58], conducted in soils heavily contaminated with crude oil in the Republic of Tatarstan, Russia, a decreasing number of bacterial gene copies was observed. The most abundant bacterial types were *Actinobacteria*, TM7 (*Saccaribacteria*), and *Bacteroidetes*.

Taxonomic analysis at genus level revealed that the control soil (C) contained 8566 ASV reads ≥1% from the phylum *Proteobacteria*. These belonged to the bacterial genera *Kaistobacter*, *Rhodoplanes*, *Ralstonia*, and *Burkholderia*; from the phylum *Actinobacteriota*, 1713 ASVs were identified, classified into the genera *Pseudonocardia*, *Streptomyces*, and *Candidatus Solibacter*. From the *Firmicutes* phylum, 291 ASVs belonging to the Bacillus genus were identified. All sorbents negatively affected the development of the most dominant bacteria in uncontaminated soil, except for the genus Streptomyces, whose ASV abundance was stimulated by agrobasalt (A) and perlite (P).

In soil contaminated with petroleum products, bacteria belonging to the genera *Rhodanobacter*, *Sphingomonas*, *Burkholderia*, *Sphingobium* and *Mycobacterium* thrived more than those in the control soil. The increasing number of ASV sequences of *Rhodanobacter* bacteria is supported by the research of Chaudhary et al. [59], who suggest a possible link between these bacteria and the higher efficiency with which they degrade diesel oil. Similarly, our study characterized the *Rhodanobacter* sp. 513602 clone, a member of the common microbiome found in both uncontaminated and contaminated soils, which stood out due to its high proportion of hydrophobic amino acids and its exceptional protein stability at −1 °C. Earlier studies by Ferguson et al. [60] provide evidence of their survival in oil-contaminated soils at low temperatures. These authors conducted analyses based on the 16S rRNA gene at the northernmost, decommissioned well, *Romulus* C-42. It was shown that *Sphingomonas* and *Rhodanobacter* bacteria, which were abundant in Arctic soils (even after 43 years), degrade polycyclic aromatic hydrocarbons, while being practically absent in control soils. Interestingly, in studies by Dillewijn et al. [55] on natural soils contaminated with diesel oil and by Song et al. [56] on soils near an active oil field did not detect bacteria from the genus *Rhodanobacter*. The role of these bacteria, which are less frequently found in both contaminated and uncontaminated environments, is therefore intriguing. Referred to as the ‘silent community’, these bacteria are marginal and difficult to detect, and their presence may play a role in biodegradation and remediation processes in contaminated soils [61].

According to Mori and Kanaly [62] cultivating soil bacteria in diesel oil revealed two pioneering bacterial genera *Sphingobium* and *Pseudomonas*. These genera’s catabolic abilities were responsible for forming specialized ecological niches. These genera utilized water-insoluble aromatic and alkane hydrocarbons, which enabled the survival of *Achromobacter* and *Cupriavidus*. The authors demonstrated that *Sphingobium* in a consortium is responsible for the initial stages of biotransformation of phenanthrene, naphthalene, and toluene. They possess nahB (bphB) genes that encode a dehydrogenase, and a methyl monooxygenase (*xylMABC*) for toluene, 1,2-dihydroxynaphthalene. They also possess genes involved in salicylic acid biodegradation (nag) and an alkane degradation marker gene (alkB; EC 1.14.15.3), as well as ferredoxin and ferredoxin reductase genes (*ahdA3A4*), as reported by Maeda et al. [63] and Liu et al. [64]. In the subsequent biodegradation of phenanthrene and naphthalene, bacteria from the genus *Pseudomonas* played a more significant role, alongside *Parvibaculum*, in the degradation of alkanes of various chain lengths, as reported by Mori and Kanaly [62]. These findings thus contribute to a better understanding of the complex ecological relationships that may evolve during the biodegradation of hydrocarbon pollutants.

#### 3.2.2. Fungi

In the study by Galitskaya et al. [58] conducted in oil-contaminated areas, the dominant fungi belonged to the phyla *Ascomycota*, *Basidiomycota*, and *Mucoromycota*. Similarly, in our research, we observed a clear dominance of *Ascomycota* and *Basidiomycota*, along with *Mortierellomycota* and *Mucoromycota*, in all analyzed soil samples. According to Costa et al. [65], Varjani and Upasani [66] and Kato et al. [67] the higher number of OTU reads in contaminated soils is most likely due to the presence of non-specific fungal degradation pathways associated with lignin-degrading enzymes, the utilization of hydrocarbons as a carbon and energy source, and a higher tolerance of fungi to hydrocarbons. In our research, the application of DO and P to the soils also altered the structure of the most abundant fungal sequence reads—*Chaetomium*, *Pseudaleuria*, and *Penicillium*—which were originally found in uncontaminated soils, while notably promoting the growth of *Penicillium*. Rani et al. [68] and Vo et al. [69] emphasize that these fungi require long-chain alkylbenzenes as a carbon and energy source for survival and are involved in mycoremediation through enzymes such as laccase and peroxidase. These fungi therefore play a key role in the biodegradation of PAHs (polycyclic aromatic hydrocarbons), particularly during the initial stages of the process. The importance of this role is confirmed by Aranda et al. [70] in their studies on fungi of the genus *Penicillium*. Our results also showed that the most stable strains were mesophilic *Penicillium* species: *Penicillium* sp. SH1167746.08, *Penicillium* sp. New.Reference, and *Penicillium* sp. SH3231803.08, which formed part of the shared mycobiome of both contaminated and uncontaminated soils. Assimilation of PAHs was also observed in *Aspergillus niger*, *Beauveria bassiana*, *Metarhizium robertsii*, and *Penicillium chrysogenum* in studies by Huarte-Bonnet et al. [71] and Ostrem Loss et al. [72].

Thus, soils contaminated with petroleum-derived substances may serve as a valuable reservoir of organisms not only capable of surviving under harsh environmental conditions but also possessing the ability to degrade toxic substances present in such soils [70,71,72]. Similarly, in our previous studies of gasoline-contaminated soil, changes were observed in the structure of the bacterial community, particularly an increase in K-strategists (slow-growing bacteria) and r-strategists (fast-growing fungi). These changes in bacterial community structure were due to the varying resistance of different microbial taxa to the toxic effects of gasoline [54].

### 3.3. The Impact of Sorbents on Soil Microbiome

The use of sorbents such as vermiculite, dolomite, perlite, or agrobasalt clearly modified the abundance of the cultured microorganisms studied. It also altered their growth rates and diversified the values of ecophysiological diversity indices. Perlite, vermiculite, and dolomite most significantly increased the abundance of organotrophic bacteria, while agrobasalt promoted to the growth of actinomycetes and fungi. In soils contaminated with diesel oil (DO) and gasoline (G), perlite reduced the number of organotrophic bacteria, whereas dolomite, vermiculite, and agrobasalt contributed to their increase. The application of all sorbents had a positive influence on the proliferation of Act (actinomycetes) and Fun (fungi) in gasoline-contaminated soil. As noted by Gul and Bora [73], Soleimani et al. [74] and Tarigholizadeh et al. [75], perlite improves water retention and soil stability. This porous, amorphous silicate material, thanks to its high specific surface area and considerable capacity for water and organic substance adsorption, may reduce the bioavailability of pollutants, thereby mitigating the toxic effects of petroleum-derived substances on the autochthonous microbiome. Moreover, it is non-toxic and chemically inert, making it safe for use. The authors also point out that perlite can create microenvironments conducive to the colonization of bacteria and fungi involved in the degradation of organic contaminants. Vermiculite has similar applications. It is characterized by a high specific surface area and cation exchange capacity within its interlayer spaces, making it an effective sorbent material for environmental remediation [32]. According to Wyszkowska et al. [26] and Wu et al. [76] dolomite also affects the physicochemical properties of soil and the activity of microorganisms responsible for the biodegradation of pollutants. The changes in the microbial community structure induced by the addition of agrobasalt can be explained by its porous structure, based on a natural clay-silicate material with high ion exchange capacity [39,77,78]. The significant potential of biochar as a sorbent material is confirmed by the studies of Wu et al. [79] and Chaudhary et al. [59].

Bacterial community analyses based on the 16S rRNA gene also indicated changes in bacterial ASVs following the application of sorbents. This was particularly evident after vermiculite was applied, as this significantly increased the number of *Rhodanobacter* ASV reads in gasoline-contaminated soil, whereas the increase was much less pronounced in diesel oil-contaminated soil. Therefore, it appears that vermiculite markedly accelerates gasoline degradation, with a slower, though still noticeable, effect observed in diesel oil-contaminated soil. The acceleration of bioremediation in petroleum-contaminated soil has also been reported by Vasilyeva et al. [14] and Erofeevskya and Aleksandrov [80]. In our earlier study Borowik et al. [25], we also found that among the tested sorbents, vermiculite proved effective in soil remediation. Thus, it should be recommended for remediation purposes in soils contaminated with diesel oil. The results of Malandrino et al. [30] and Topka et al. [31] and Brião et al. [32] and Szerement et al. [33] indicate that vermiculite, as a natural clay mineral with a well-developed layered structure, is effective in adsorbing organic compounds and heavy metal ions. Importantly, the chemical and physical stability of vermiculite under environmental conditions allows for its long-term application as a soil and water purification support material, as it significantly reduces the levels of polycyclic aromatic hydrocarbons (PAHs). In light of the obtained data, it can therefore be concluded that vermiculite has substantial potential in the long-term sequestration of contaminants in soil environments.

The results of our study indicate that vermiculite, perlite and agrobasalt create more favorable conditions for the proliferation of *Rhodanobacter*, *Mycobacterium* and *Bacillus* in gasoline-contaminated soil. Additionally, perlite stimulated the growth of *Planifilum* and *Microbacterium* in the presence of agrobasalt, and of *Achromobacter*, *Sphingobium*, *Streptomyces* and *Devosia* in the presence of dolomite. Like vermiculite, dolomite demonstrates significant potential as a mineral sorbent in the remediation of soils contaminated with petroleum-derived products such as diesel oil and gasoline. Giagnoni et al. [81] attributed a beneficial role to this sorbent in the phytoremediation of soil. According to Wyszkowska et al. [26] and Wu et al. [76] dolomite serves as a source of calcium and magnesium and neutralizes soil acidity, thereby optimizing pH conditions for microbial activity. Furthermore, it increases the cation exchange capacity (CEC) and organic carbon content. Wu et al. [76] ascribes the positive effect of dolomite to its ability to rapidly disperse in soil.

In our study, all of the tested sorbents reduced the number of ASV reads of *Rhodanobacter*, *Parvibaculum*, *Sphingomonas*, *Burkholderia* and *Achromobacter* in diesel-contaminated (DO) soils, with dolomite having the strongest effect. In contrast, in gasoline-contaminated soils, the ASV count of *Rhodanobacter* increased only after dolomite application. Dolomite is a sedimentary rock mainly composed of calcium and magnesium carbonates, which can significantly affect soil pH [16,24,25,26,39,54]. Bucheli et al. [82], explain the increase in soil pH by demonstrating that a higher pH reduces the rate of PAH sorption. This occurs because under low pH conditions, the functional groups of soil organic matter are protonated and thus less hydrophilic, favoring better PAH sorption. A similar conclusion was drawn by [83] who reported that petroleum hydrocarbons can alter soil pH and nutrient availability, which may affect soil chemistry.

Among the analyzed sorbents, vermiculite had a particularly significant effect on fungal abundance. Under uncontaminated soil conditions, it favored the development of *Chaetomium*, *Fusarium*, and *Mucor*, whereas in soils contaminated with diesel oil (DO) and gasoline (G), it supported the growth of *Penicillium* and *Fusarium*. According to studies by Wu et al. [84] vermiculite also showed the greatest effectiveness in improving soil environmental properties. Similarly, perlite, as reported by Moscoso et al. [85] increased the population of microorganisms capable of releasing phosphorus from insoluble mineral compounds and fixing nitrogen. Agrobasalt, according to Wu et al. [79] positively influences nutrient cycling in the soil and enhances their biological availability. These findings are consistent with our study results, in which perlite and agrobasalt stimulated the growth of *Cladorrhinum*, a plant growth-promoting fungi [86], in uncontaminated soils. Additionally, agrobasalt promoted the development of *Humicola* fungi, which have a high potential to suppress pathogen invasion and disease development [87]. In soils contaminated with gasoline, agrobasalt also promoted the growth of *Penicillium*. In soils contaminated with DO and G, perlite supported the growth of fungi from the *Gymnostellatospora* genus (which is closely related to myxotrichaceous fungi, such as *Pseudogymnoascus*, *Geomyces* and *Gymnostellatospora*, that are most frequently isolated from peatlands). The abundance of these fungi suggests that they may be important saprotrophs in the ecosystem [88].

Sorbents, as carriers of microorganisms, not only bacteria but also fungi, should be used in the bioremediation of contaminated soils.

The results of the conducted research confirm the effectiveness of mineral sorbents such as vermiculite, dolomite, perlite, and agrobasalt in reducing the bioavailability and toxicity of polycyclic aromatic hydrocarbons (PAHs) in the soil environment. These sorbents mainly act through adsorption, whereby PAH molecules bind to the sorbent surface or within its porous structures. This decreases the solubility and mobility of these compounds in the soil solution. Consequently, the quantity of compounds available to microorganisms decreases, alleviating environmental stress and supporting the regeneration of the autochtonous microbiome [89,90,91].

### 3.4. Implications of Protein Property Predictions Derived from Nucleotide Sequence Data

The structural stability of biomolecules, such as RNA and proteins, is crucial for maintaining the metabolic functions of microorganisms in soil. In a contaminated environment, these structures are often destabilized by oxidative stress or disturbances in ion homeostasis. This can disrupt essential processes such as translation and transcription [13]. The difference observed in the predicted stability of RNA structures in uncontaminated and contaminated soil samples may indicate the adaptive mechanisms, selection and the ability of these microorganisms to maintain molecular integrity in harsh, unfavorable conditions. Such changes may directly impact the dynamics of microbial communities and their functional potential.

The research shows that, in response to diesel oil (DO) and gasoline (G), the levels of Ala (DO, G) and Thr (G) increase in bacteria (see Table 1). The increased production of alanine in microorganisms under stressful conditions is due to the activation of adaptive pathways, in which alanine maintains energy balance by associating with the glycolysis process. Thus, this compound performs a dual function: it enables the conversion of pyruvate and the regeneration of NAD^+^, thereby performing a detoxification function; and it supports the survival of bacteria under stressful conditions by performing a metabolic function [92]. Conversely, elevated threonine levels may indicate the activation of anabolic pathways and the adaptation of cell membranes to hydrophobic contaminants [93]. A decrease in Cys and Gly values in bacterial cells was also observed. This can be explained by the use of these amino acids in the synthesis of glutathione (GSH), one of the most important antioxidants which protects cells from oxidative stress induced by the analyzed organic pollutants [94]. Therefore, reduced levels of cysteine and glycine indicate a weakening of the cell’s protective abilities and severe oxidative stress. The high glycine content in the predicted structure of bacterial proteins may also be due to the fact that, of the 20 most common proteogenic amino acids, glycine is the only achiral amino acid with two hydrogen atoms attached to the central carbon (i.e., the side chain R = H), which imparts high conformational flexibility to proteins and explains their widespread occurrence and structural stability in bacterial proteomes [95]. In turn, the increase in these amino acids observed in fungi (Table 3) indicates the activation of a detoxification mechanism based on the intensification of glutathione (GSH) synthesis [96].

The predictability of minimum free energy (MFE) enables the calculation of the secondary structure of RNA that is most energetically favorable, and thus most likely [97,98]. Bioinformatic algorithms use thermodynamic models of RNA molecule folding to find a conformation characterized by the lowest possible Gibbs free energy (ΔG) value. A lower energy system indicates greater thermodynamic stability of a given arrangement of nitrogen bases, suggesting that a given secondary structure is crucial for biological activity. In our study, bacteria forming a common microbiome in gasoline-contaminated and uncontaminated soils (G, C) exhibited greater protein structural stability, as evidenced by a lower free energy value (MFE = −151.30). In contrast, the lowest values (MFE = −88.30) and the most stable sequences occurred in the shared microbiome of contaminated soils (G, DO) in the case of fungi. This suggests that microorganisms inhabiting more complex and toxic environments can evolve molecular mechanisms that offer greater resilience to environmental stress [99], while demonstrating enhanced potential for the remediation of contaminated soils [100,101]. They possess more effective regulatory systems, superior DNA repair mechanisms and more stable proteins [99,102].

In the study by Aarti et al. [97], the energy structure of the identified bacterial and fungal strains changed with the GC content of the sequence, affecting its secondary structure. Computational methods that predict mRNA secondary structures and minimum free energy (MFE) are important for the effective expression of proteins derived from foreign organisms [103].

The results obtained indicate the significant potential of using the soil microbiome as a sensitive indicator of the effectiveness of remediation processes in soils contaminated with petroleum compounds. It was found that, regardless of the type of sorbent and petroleum product used, the highest stability was exhibited by the bacterial strains *Pseudonocardia* sp. 2595164, *Ralstonia* sp., *Streptomyces* sp. 1082846, and *Bacillus* sp. 1051517 (isolated from soils G and C). Among the fungal strains, *Penicillium* sp. New. Reference (G and DO), *Talaromyces* clone SH1676308.08, *Penicillium* sp. New. Reference, and *Actinomucor* sp. SH1621089.08 (G) demonstrated the highest stability. Their presence and relative resistance to stress caused by petroleum products may indicate their adaptation to contaminated environments. This may indicate their potential usefulness as bioindicators and microorganisms supporting the remediation of such soils. Changes in the structure and diversity of microbial communities can provide information on the progress or failure of remediation processes earlier than traditional chemical analyses can. Additionally, differences in the impact of individual sorbents on the soil microbiome under pressure from the studied pollutants may aid in the more effective selection depending on the type of contamination, which is crucial for soil protection. Thus, the results of this study could inform the more sustainable management of soils contaminated with various organic compounds.

## 4. Materials and Methods

### 4.1. Characterization of Soil Material, Sorbents, and Petroleum-Derived Substances

The study was conducted on soil samples collected from the 0–20 cm layer in Tomaszkowo near Olsztyn, north-eastern Poland (53.710° N, 20.4320° E). The soil was classified as Eutric Cambisol. It had a loamy sand texture. The content (%) was as follows: sand—73.46; silt—24.29; clay—2.25. Content per 1 kg of soil dry matter: C_org_—6.95 g; N_Total_ —1.06 g, HAC—34.56 mmol^(+)^, EBC—44.82 mmol^(+)^, CEC—79.38 mmol^(+)^, BS—56.46 and pH_KCl_ 4.2.

**Figure 7:**
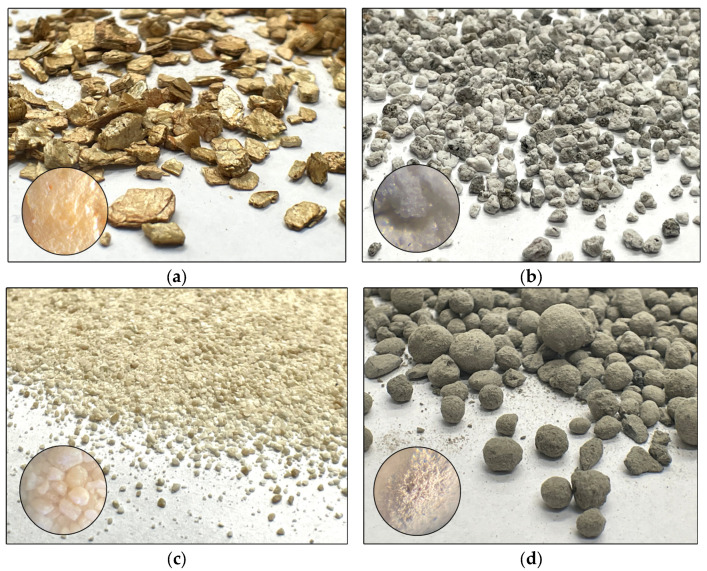
Sorbents used in the experiment: (**a**) vermiculite; (**b**) perlite; (**c**) dolomite; (**d**) agrobasalt. Photographs were taken with a digital camera at 2× magnification and under a microscope at 40× magnification.

The sorbents (Figure 7) used in the experiment were applied at rates of 0 and 10 g kg^−1^ of dry matter soil. Dolomite and perlite were supplied by Biovita Sp. z o.o., Tenczynek, Poland, while vermiculite and agrobasalt were provided by Sobex, Drezenko, Poland. The characteristics of the sorbents have been described in our previous studies [25,26,39].

Diesel oil VERVA (DO) for diesel engines and unleaded gasoline 95 (G) were purchased in June 2023 (grade B with CFPP, max. 0 °C) from a PKN Orlen station (Poland). The density of DO ranged from 0.820 to 0.845 g cm^−3^, with a sulfur content of up to 10 mg kg^−1^. The density of G ranged from 0.720 to 0.775 g cm^−3^, with a sulfur content of up to 10 mg kg^−1^. The detailed characteristics of the soil, petroleum-derived substances, and sorbents have been described in our previous studies [25,39].

### 4.2. Experimental Design

The research experiment was conducted in a split-plot design with four replications, under controlled conditions in a vegetation hall. The experiment was carried out in pots with the following dimensions: 15 cm (ϕ bottom) × 20 cm (ϕ top) × 17 cm (height). The first experimental factor was the type of petroleum-derived substance: control (C), diesel oil (DO), and unleaded petrol 95 (G). The second factor was the type of sorbent: control (C), vermiculite (V), dolomite (D), perlite (P), and agrobasalt (A).

After transporting the soil to the vegetation hall, the first step was the application of basic fertilization with N, P, K, and Mg, was applied uniformly across all treatments, amounting to, in mg per 1 kg of dry soil: N—225, P—50, K—150, and Mg—20. Nutrients were applied in the form of aqueous solutions of CO(NH_2_)_2_, KH_2_PO_4_, KCl, and MgSO_4_·7H_2_O. Subsequently, petroleum-derived products were applied to the appropriate treatments at a rate of 16 cm^3^ kg^−1^ dry soil, and sorbents were added at a rate of 10 g kg^−1^ soil.

The test plant was maize (*Zea mays*), cultivar DS1897B, sown at a density of 4 plants per pot. Harvest was carried out at growth stage BBCH 59, on the 60th day of cultivation. Throughout the experimental period, soil moisture was maintained at 60%.

### 4.3. Cultivable Microorganisms

The abundance of organotrophic bacteria (Org), actinomycetes (Act), and fungi (Fun) was determined using the plate method with serial dilutions. The following media were used for cultivation: Bunt and Rovira medium [104] for Org, Küstera and Williamsa [105] for Act and Martina [106] for fungi. Soil samples were suspended in physiological saline solution (0.85% NaCl), and microorganisms were isolated using the deep plating method (four replicates). Incubation was carried out for 10 days at 28 °C (Selecta Incudigit, Barcelona, Spain). Results were expressed as CFU kg^−1^ of dry soil.

### 4.4. Non-Cultivable Microorganisms

Genomic DNA was isolated using the Fast DNA Spin Kit for Soil (MP Biomedicals, Inc., Irvine, CA, USA). The DNA was suspended in nuclease-free water and purified using the Anti-Inhibitor Kit (A&A Biotechnology, Gdańsk, Poland). The concentration of DNA in the isolate was verified by fluorometric measurement using the Quant-iT™ PicoGreen™ dsDNA Assay Kit (Thermo Fisher Scientific, Waltham, MA, USA). The presence, quality and purity of the bacterial DNA were confirmed by microfluidic electrophoresis.

Sequencing of the 16S rRNA gene fragment and the ITS region was performed targeting the hypervariable V3–V4 region using the Illumina MiSeq sequencer and MiSeq Reporter (MSR) software v2.6 (Genomed S.A., Warsaw, Poland). Amplification of the selected region for bacterial and archaeal populations was carried out using primers 341F (5′-CCTACGGGNGGCWGCAG-3′) and 785R (5′-GACTACHVGGGTATCTAATCC-3′). Bioinformatic analysis for read classification was conducted using the QIIME 2 software package based on the GreenGenes v13_8 reference sequence database.

Metagenomic analysis of fungal populations was performed based on the hypervariable ITS1 (5′-GAACCWGCGGARGGATCA-3′) and 5.8S (5′-CGCTGCGTTCTTCATCG-3′) regions. Bioinformatic analysis was carried out using QIIME software based on the UNITE v8.2 reference sequence database. The use of ASVs for bacteria was dictated by its high accuracy and ability to distinguish precisely between individual sequence variants and taxonomic diversity. For fungi, we used Operational Taxonomic Units (OTUs) because grouping them based on a 97% similarity threshold ensures greater stability of results by taking into account sequencing errors and reducing the risk of false positives [107,108]. The microbial sequences have been deposited in the National Center for Biotechnology Information (NCBI) database and are available via the following links: Prokaryotic 16S rRNA: https://www.ncbi.nlm.nih.gov/nuccore/?term=PQ531260:PQ532934[accn] (accessed on 3 November 2024); Eukaryotic Nuclear rRNA/ITS: PQ741026-PQ741430 Eukaryotic Nuclear rRNA/ITS: https://www.ncbi.nlm.nih.gov/nuccore/?term=PQ741026:PQ741430[accn] (accessed on 17 December 2024).

### 4.5. Data Analysis and Statistical Processing

The collected data, such as colony counts (CFU), colony development index (CD), and ecophysiological diversity indices (EP) characterizing soil microorganisms, were statistically processed using Statistica 13.3 [109] and TBtools-IIv2.310 [110]. Analyses were performed at a significance level of α = 0.05. Microbial abundance data were used to calculate colony development (CD) and ecophysiological diversity (EP) indices following formulas proposed by Sarathchandra et al. [111] and De Leij [112].

Metagenomic data were presented after excluding ASVs and ITS sequences representing less than 1% of the total number of ASVs or ITS sequences. Statistical analysis and visualization of the data were carried out using R software v1.2.5033 (Boston, MA, USA) with R v3.6.2 [113]. Metagenomic data were used to calculate diversity indices including Shannon–Weaver (H), Simpson (D), Margalef (Dm), Richness (R), and Pielou’s evenness (J), as described by Jiang et al. [114] and Yang et al. [115]. Diversity indices were calculated separately for bacteria and fungi using Microsoft Excel^®^ for Microsoft 365 MSO version 2206 [116].

Furthermore, the sequences obtained from metagenomic analysis were thoroughly analyzed using the ProtParam tool (https://web.expasy.org/protparam/, (accessed on 27 May 2025) available on the ExPASy portal [117]. The analysis included the number of amino acids, molecular weight, and instability index. Based on the presence of aliphatic amino acid residues in the proteins, the aliphatic index was calculated to estimate protein thermostability. The grand average of hydropathicity (GRAVY) was also determined.

To confirm protein stability, RNAfold was used, which applies the minimum free energy (MFE) algorithm to predict the most probable RNA secondary structure in thermodynamic equilibrium. Analyses were performed at two temperature ranges: the average winter temperature (−1 °C) typical for northern Poland from December to February, and the average summer temperature (17 °C) from June to August. RNA parameters [118] were computed using the Turner 2004 model via the RNAfold web tool (http://rna.tbi.univie.ac.at//cgi-bin/RNAWebSuite/RNAfold.cgi, (accessed on 27 May 2025). A phylogenetic tree was constructed using MEGA-11 software by the Maximum Likelihood (ML) method.

## 5. Conclusions

Diesel oil induced greater changes in microbial diversity than gasoline. DO stimulated the proliferation of cultivable microorganisms, whereas unleaded gasoline inhibited it. The contrasting effects of gasoline and diesel fuel on cultured microorganisms are due to their different chemical compositions. Both petroleum products increased the abundance of ASVs affiliated with *Rhodanobacter*, *Sphingomonas*, *Burkholderia*, *Sphingobium*, and *Mycobacterium*, as well as fungi of the genus *Penicillium*. However, they had a negative effect on *Kaistobacter*, *Rhodoplanes*, and *Ralstonia*, and the fungi *Chaetomium*, *Pseudaleuria*, and *Mortierella*. The core microbiome included clones *Rhodanobacter* sp. 513602, *Penicillium* sp. SH1167746.08, *Penicillium* sp. New. Reference, and *Penicillium* sp. SH3231803.08. The presence of these microorganisms as native microbiome members indicates their versatility and adaptive capacity in response to changing environmental conditions. Therefore, strains among these bacteria and fungi should be considered for bioaugmentation of soils contaminated with petroleum hydrocarbons. Nevertheless, better results in bioaugmentation can be achieved by using bacteria because their proteins are more stable than fungal proteins. Even better results may be achieved when bioaugmentation is supported with mineral sorbents. The application of mineral sorbents, such as vermiculite, dolomite, perlite, and agrobasalt, helped restore the balance of soil microbiomes destabilized by petroleum-derived products. The addition of sorbents to contaminated soil contributed to changes in the composition of bacterial and fungal communities. All of the sorbents enhanced the growth of organotrophic bacteria (Org) and fungi (Fun) in diesel oil (DO)-contaminated soil, as well as actinobacteria (Act) and fungi in soil polluted with unleaded gasoline (G). Vermiculite (V) and agrobasalt (A) had the most beneficial effects on cultivable microorganisms. Mineral sorbents reduced the extent of microbiome disturbances, as assessed based on non-cultivable bacteria and fungi, caused by DO and G. Thus, the stability of biomolecular structures can serve as an indicator of microbial resistance and as a valuable tool for assessing pollutant impacts on soil microbial ecosystems.

Our research has proven that in order to effectively manage the remediation of soil contaminated with petroleum products, vermiculite and agrobasalt should be used in combination with bioaugmentation of bacterial isolates from the genera *Rhodonobacter*, *Sphingomonas*, *Burkholderia*, *Sphingobium*, and *Mycobacterium*.

## Figures and Tables

**Table 1:** Predicted protein properties based on nucleotide sequences of bacterial clones identified in soil from individual treatments. Average results calculated based on data from Table S1.

Treatment	Amino Acid Composition, %				Molecular Weight (kDa)	Instability Index	Aliphatic Index	GRAVY
	Ala	Cys	Gly	Thr				
C	24.86	21.27	34.76	19.11	32.52	40.89	24.85	0.71
DO	26.11	20.69	33.58	19.62	32.96	40.38	26.11	0.72
G	27.05	20.05	32.70	20.30	32.76	40.66	27.02	0.71
G, DO	25.83	20.73	33.90	19.53	32.31	42.18	25.81	0.71
G, C	24.83	21.28	35.10	18.78	32.83	40.99	24.83	0.71
G, DO, C	26.20	20.10	34.20	19.40	33.57	38.62	26.23	0.70
average	25.81	20.69	34.04	19.46	32.83	40.62	25.81	0.71

GRAVY—grand average of hydropathicity; C—bacteria identified in uncontaminated soil; DO—bacteria identified in diesel oil-contaminated soil; G—bacteria identified in gasoline-contaminated soil; G, DO—bacteria shared between gasoline- and diesel oil-contaminated soils; G, C—bacteria shared between gasoline-contaminated and uncontaminated soils; G, DO, C—bacteria shared among gasoline-contaminated, diesel oil-contaminated, and uncontaminated soils.

**Table 2:** Minimum free energy (MFE), community diversity (pz), and predicted RNA secondary structure based on bacterial nucleotide sequences at two temperature ranges. Mean values calculated from the data presented in Table S2.

Treatment	MFE	Diversity (pz)	The Secondary Structure	MFE	Diversity (pz)	The Secondary Structure
	Temperature −1 °C			Temperature 17 °C		
C	−150.77	64.14	−138.14	−217.27	53.74	−204.75
DO	−146.10	75.98	−126.84	−211.97	53.72	−197.58
G	−136.85	94.73	−99.10	−200.95	59.44	−194.02
G, DO	−144.55	79.26	−124.95	−209.78	60.55	−188.39
G, C	−151.30	82.57	−125.78	−218.26	63.03	−199.13
G, DO, C	−135.70	89.09	−106.70	−199.55	69.20	−198.02
average	−144.21	80.96	−120.25	−209.63	59.95	−196.98

C—bacteria identified in uncontaminated soil; DO—bacteria identified in diesel oil-contaminated soil; G—bacteria identified in gasoline-contaminated soil; G, DO—bacteria shared between gasoline- and diesel oil-contaminated soils; G, C—bacteria shared between gasoline-contaminated and uncontaminated soils; G, DO, C—bacteria shared among gasoline-contaminated, diesel oil-contaminated, and uncontaminated soils.

**Table 3:** Predicted protein properties based on nucleotide sequences of fungal clones identified in soil from each treatment. Average results calculated from data in Table S3.

Treatment	Amino Acid Composition, %				Molecular Weight (kDa)	Instability Index	Aliphatic Index	GRAVY
	Ala	Cys	Gly	Thr				
C	28.51	23.91	20.48	26.96	20.29	55.15	28.52	0.84
DO	21.80	29.45	26.40	22.30	21.56	55.54	21.82	0.87
G	23.13	27.67	26.10	23.10	22.75	51.00	23.14	0.84
G, DO	20.90	30.70	27.00	21.30	20.40	50.29	30.90	0.89
G, C	25.33	26.98	23.13	24.52	20.92	50.29	30.90	0.89
G, DO, C	21.33	31.63	26.47	20.53	20.55	47.19	21.34	0.93
average	23.50	28.39	24.93	23.12	21.08	51.58	26.10	0.88

GRAVY—grand average of hydropathicity; C—fungi identified in uncontaminated soil, DO—fungi identified in soil contaminated with diesel oil, G—fungi identified in soil contaminated with gasoline, G, DO—fungi common to soil contaminated with gasoline and diesel oil, G, C—fungi common to soil contaminated with gasoline and uncontaminated soil, G, DO, C—fungi common to soil contaminated with gasoline, diesel oil, and uncontaminated soil.

**Table 4:** Minimal free energy (MFE), diversity of pz clusters, and predicted RNA secondary structure based on nucleotide sequences of fungi at two temperature ranges. Average results calculated from data in Table S4.

Treatment	MFE	Diversity (pz)	The Secondary Structure	MFE	Diversity (pz)	The Secondary Structure
	Temperature −1 °C			Temperature 17 °C		
C	−60.63	43.84	−54.40	−93.46	39.34	−81.14
DO	−84.30	50.55	−75.40	−121.44	45.61	−109.17
G	−87.57	57.37	−80.07	−129.76	35.18	−122.79
G, DO	−88.30	59.64	−64.70	−125.27	49.78	−105.72
G, C	−72.67	54.97	−56.35	−108.11	47.69	−95.90
G, DO, C	−85.13	52.49	−67.97	−120.76	48.75	−104.82
average	−79.77	53.14	−66.48	−116.47	44.39	−103.26

C—fungi identified in uncontaminated soil; DO—fungi identified in soil contaminated with diesel oil; G—fungi identified in soil contaminated with gasoline; G, DO—fungi common to soil contaminated with gasoline and diesel oil; G, C—fungi common to soil contaminated with gasoline and uncontaminated soil; G, DO, C—fungi common to soil contaminated with gasoline, diesel oil, and uncontaminated soil.

## Data Availability

The original contributions presented in this study are included in the article and Supplementary Materials. Further inquiries can be directed to the corresponding author.

## Supplementary Materials

The following supporting information can be downloaded at: https://www.mdpi.com/article/10.3390/ijms26136491/s1.
