# Supplementary material for: Regulation of the Microbiome in Soil Contaminated with Diesel Oil and Gasoline
Source: Int J Mol Sci. 2025 Jul 5;26(13):6491. doi: 10.3390/ijms26136491 (PMC12250306; doi:10.3390/ijms26136491)
Supplement: Supplementary file 1 [file ijms-26-06491-s001.zip › ijms-3715541-supplementary.pdf]

Supplementary Material

Regulation of the microbiome in soil contaminated with diesel oil and gasoline

Agata Borowik, Jadwiga Wyszkowska*, Magdalena Zaborowska and Jan Kucharski

[1] Department of Soil Science and Microbiology, Faculty of Agriculture and Forestry, University of Warmia and Mazury in Olsztyn, 10–719 Olsztyn, Poland; agata.borowik@uwm.edu.pl; m.zaborowska@uwm.edu.pl; jan.kucharski@uwm.edu.pl

* Correspondence jadwiga.wyszkowska@uwm.edu.pl

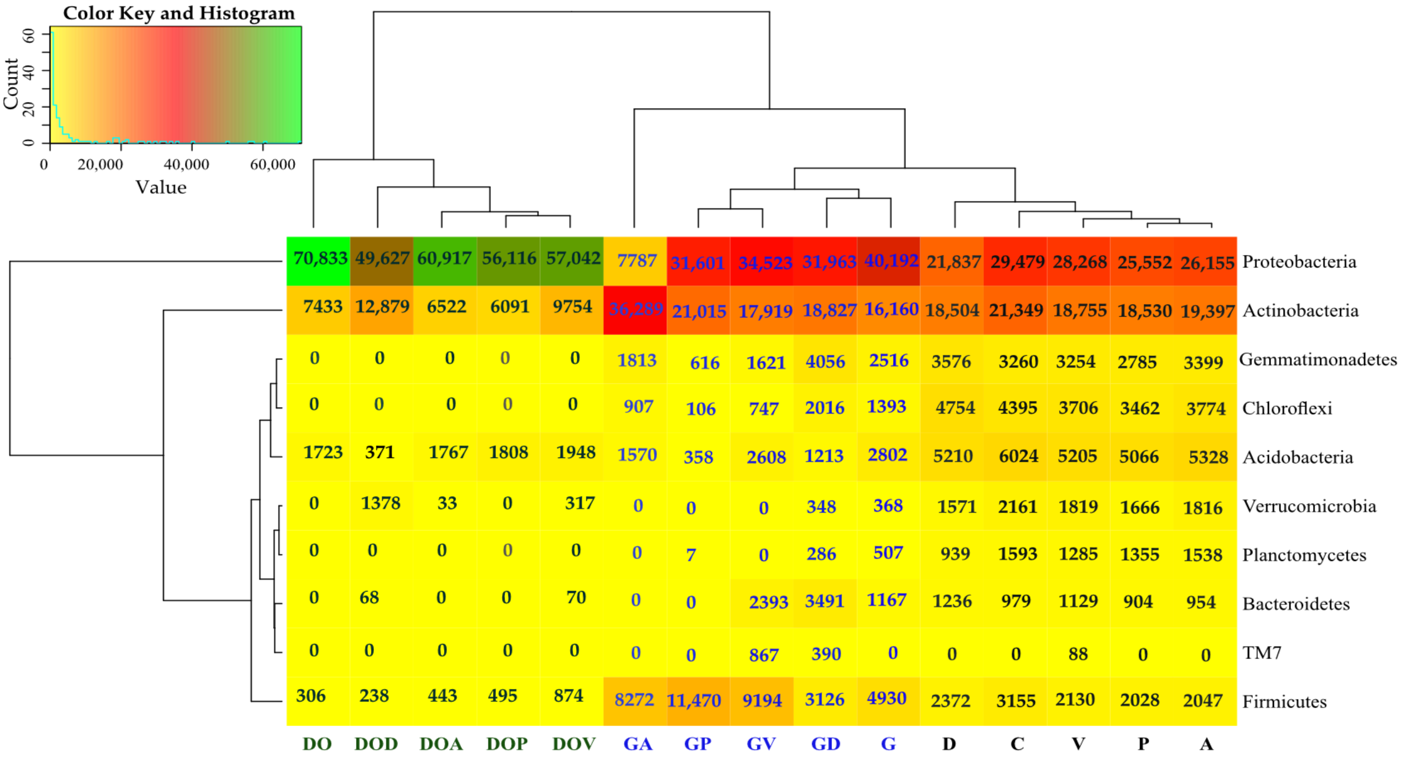

Figure S1. Effect of sorbents and petroleum-derived products on the abundance (ASV ≥ 1%) of bacterial phyla in soil. C – uncontaminated soil; V – uncontaminated soil with vermiculite; D – uncontaminated soil with dolomite; P – uncontaminated soil with perlite; A – uncontaminated soil with agrobasalt; G – soil contaminated with gasoline; GV – gasoline-contaminated soil with vermiculite; GD – gasoline-contaminated soil with dolomite; GP – gasoline-contaminated soil with perlite; GA – gasoline-contaminated soil with agrobasalt; DO – soil contaminated with diesel oil; DOV – diesel oil-contaminated soil with vermiculite; DOD – diesel oil-contaminated soil with dolomite; DOP – diesel oil-contaminated soil with perlite; DOA – diesel oil-contaminated soil with agrobasalt.

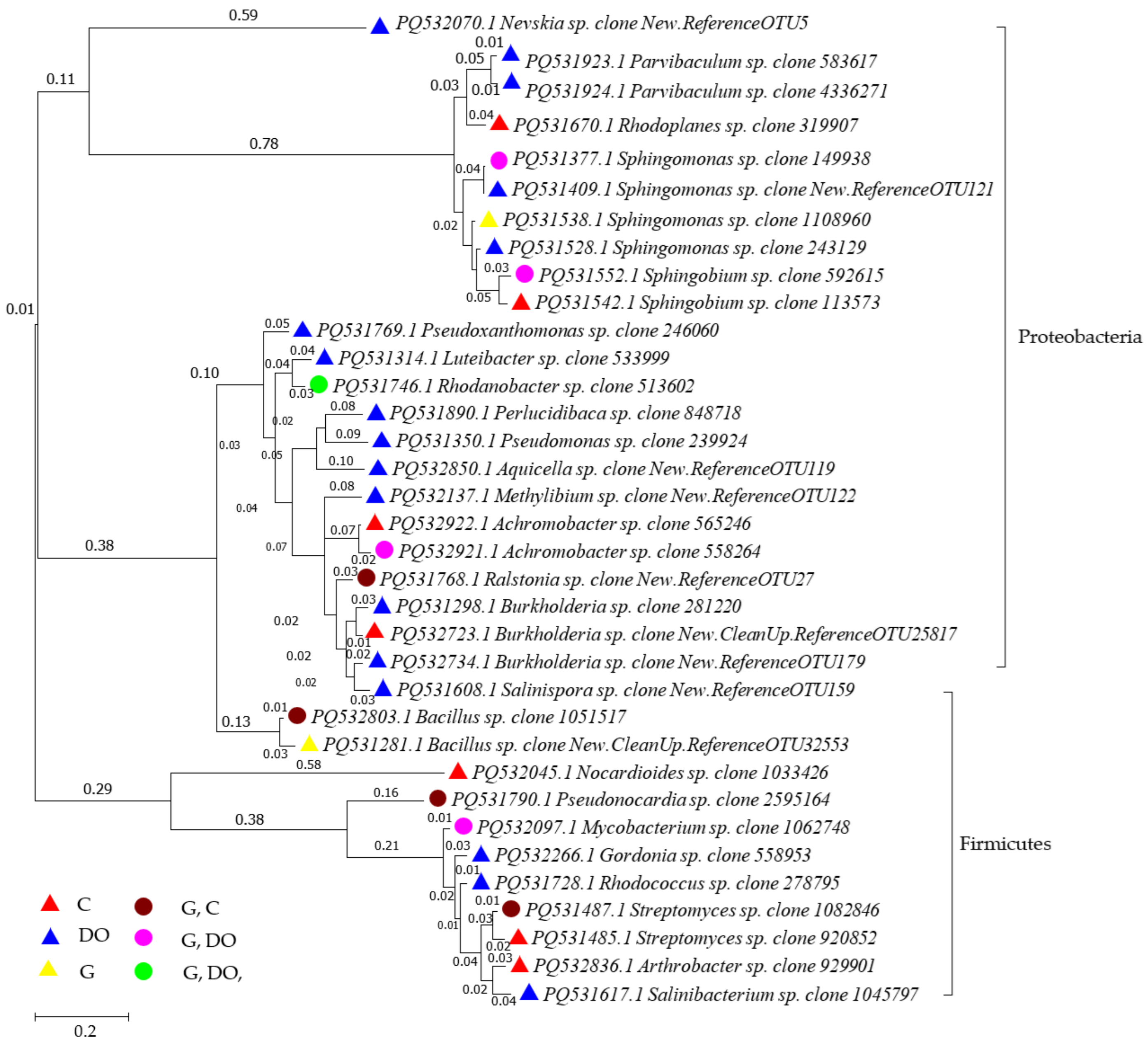

Figure S2. Phylogenetic tree showing the sequences of bacteria capable of surviving in uncontaminated soil and in soil contaminated with diesel oil and gasoline. C – uncontaminated soil; DO – soil contaminated with diesel oil; G – soil contaminated with gasoline.

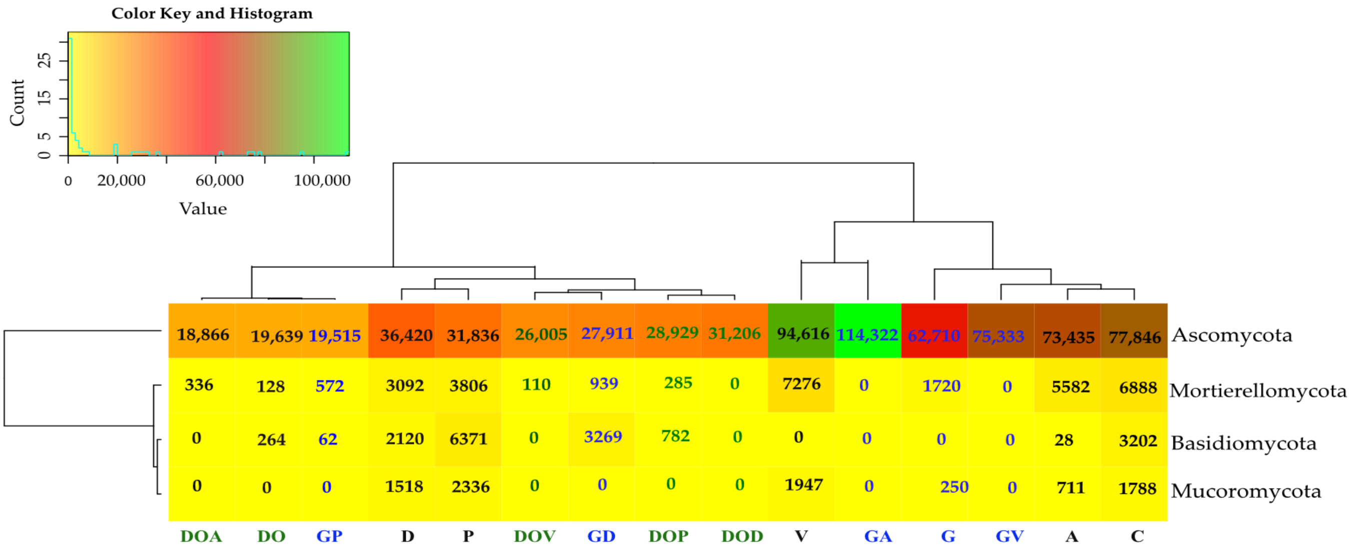

Figure S3. Effect of sorbents and petroleum-derived products on the abundance (ASV ≥1%) of fungal types in soil. C – uncontaminated soil; V – uncontaminated soil with vermiculite; D – uncontaminated soil with dolomite; P – uncontaminated soil with perlite; A – uncontaminated soil with agrobasalt; G – soil contaminated with gasoline; GV – gasoline-contaminated soil with vermiculite; GD – gasoline-contaminated soil with dolomite; GP – gasoline-contaminated soil with perlite; GA – gasoline-contaminated soil with agrobasalt; DO – soil contaminated with diesel oil; DOV – diesel oil-contaminated soil with vermiculite; DOD – diesel oil-contaminated soil with dolomite; DOP – diesel oil-contaminated soil with perlite; DOA – diesel oil-contaminated soil with agrobasalt.

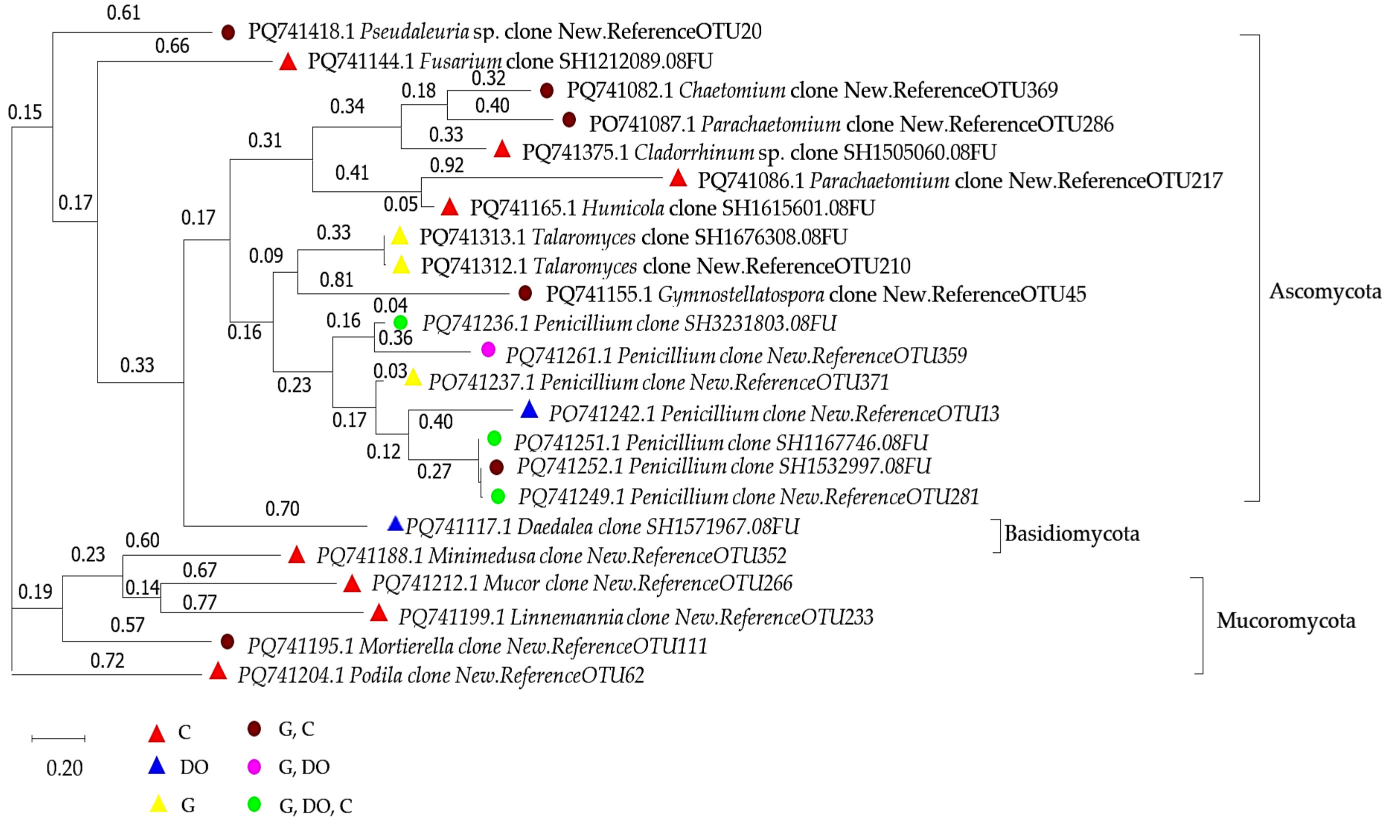

Figure S4. Phylogenetic tree showing the sequences of fungi capable of surviving in uncontaminated soils and those contaminated with diesel oil and gasoline. C – uncontaminated soil; DO – soil contaminated with diesel oil; G – soil contaminated with gasoline.

Table S1. Predicted protein properties calculated based on nucleotide sequences of bacterial clones identified in soil from individual treatments. The calculations were performed using the ProtParam database program (https://web.expasy.org/protparam/) on the ExPasy portal.

Treatment	Bacterial clones	Amino acid structure %				Molecular weight (kDa)	Instability index	Aliphatic index	GRAVY
		Ala	Cys	Gly	Thr				
C	*Arthrobacter* sp. 929901	24.60	21.10	35.90	18.40	31.91	40.82	24.57	0.70
	Sphingobium sp. 113573	26.10	20.10	33.80	19.90	31.68	36.62	26.12	0.70
	Streptomyces sp. 920852	23.80	21.60	36.40	18.20	31.91	39.97	23.83	0.70
	Nocardioides sp. 1033426	22.40	20.90	36.80	20.00	32.72	39.38	22.36	0.64
	Burkholderia sp. New.Reference	25.50	21.30	34.00	19.20	33.71	42.41	25.53	0.72
	Achromobacter sp. 565246	27.20	21.50	31.60	19.70	33.95	44.18	27.17	0.76
	Rhodoplanes sp. 319907	24.40	22.40	34.80	18.40	31.74	42.87	24.38	0.73
DO	*Sphingomonas* sp. 243129	26.60	19.40	32.80	21.10	31.79	37.02	26.62	0.69
	Burkholderia sp. 281220	26.00	21.30	33.50	19.20	33.74	41.14	26.00	0.73
	Parvibaculum sp. 583617	25.90	21.40	33.10	19.70	31.86	40.06	25.87	0.73
	Luteibacter sp. 533999	25.80	20.80	34.20	19.20	33.64	37.03	25.76	0.71
	Salinibacterium sp. 1045797	25.60	21.10	34.90	18.40	31.96	43.39	25.55	0.72
	Rhodococcus sp. 278795	24.60	21.60	35.10	18.70	32.04	46.26	24.57	0.71
	Burkholderia sp. New.Reference	26.00	21.10	33.50	19.40	33.74	37.42	26.00	0.73
	Methylibium sp. New.Reference	26.90	21.80	31.60	19.70	33.98	44.67	26.93	0.77
	Nevskia sp. New.Reference	26.10	20.90	33.10	20.00	33.73	40.61	26.26	0.72
	Sphingomonas sp. New.Reference	26.60	19.70	33.80	19.90	31.62	39.87	26.62	0.70
	Gordonia sp. 558953	24.10	21.60	35.40	18.90	32.06	45.35	24.08	0.70
	Parvibaculum sp. 4336271	25.90	21.40	33.30	19.40	31.81	40.72	25.87	0.73
	Pseudoxanthomonas sp. 246060	26.20	19.90	33.00	20.80	33.79	40.55	26.23	0.69
	Pseudomonas sp. 239924	27.40	19.20	32.10	21.30	33.81	41.33	27.40	0.70
	Salinispora sp. New.Reference	26.20	21.80	33.70	18.30	33.67	38.65	26.23	0.75
	Aquicella sp. New.Reference	27.20	18.70	34.70	19.40	33.35	33.80	27.17	0.68
	Perlucidibaca sp. 848718	26.70	20.10	33.00	20.10	33.73	38.61	26.70	0.71
G	*Sphingomonas* sp. 1108960	26.90	19.70	32.60	20.90	31.81	38.55	26.87	0.70
	Bacillus sp. New.Reference	27.20	20.40	32.80	19.70	33.72	42.76	27.17	0.73
G, DO	*Sphingomonas* sp. New.Reference	26.40	19.90	33.80	19.90	31.65	40.09	26.37	0.70
	Achromobacter sp. 558264	27.20	21.50	31.90	19.40	33.90	42.58	27.17	0.76
	Sphingobium sp. 592615	25.90	20.40	33.80	19.90	31.72	39.50	25.87	0.70
	Mycobacterium sp. 1062748	23.80	21.10	36.10	18.90	31.95	46.56	23.83	0.68

G, C	*Pseudonocardia* sp. 2595164	23.00	22.10	36.80	18.10	32.02	44.59	23.04	0.69
	Ralstonia sp. New.Reference	26.50	20.10	33.30	20.10	33.72	36.53	26.46	0.71
	Streptomyces sp. 1082846	23.60	21.60	36.60	18.20	31.90	39.97	23.59	0.69
	Bacillus sp. 1051517	26.20	21.30	33.70	18.70	33.67	42.88	26.23	0.74
G, DO, C	*Rhodanobacter* sp. 513602	26.20	20.10	34.20	19.40	33.57	38.62	26.23	0.70

GRAVY – grand average of hydropathicity; C – bacteria identified in uncontaminated soil; DO – bacteria identified in soil contaminated with diesel fuel; G – bacteria identified in soil contaminated with gasoline; G, DO – bacteria common to soil contaminated with gasoline and diesel fuel; G, C – bacteria common to gasoline-contaminated and uncontaminated soil; G, DO, C – bacteria common to gasoline-contaminated, diesel-contaminated, and uncontaminated soil.

Table S2. Minimum free energy (MFE), diversity of pz complexes, and predictable secondary structure of RNA based on bacterial nucleotide sequences in two temperature ranges calculated using the Turner model (2024). The calculations were performed using the RNAfold program (http://rna.tbi.univie.ac.at//cgi-bin/RNAWebSuite/RNAfold.cgi).

Treatment	Bacterial clones	MFE	Diversity (pz)	The secondary structure	MFE	Diversity (pz)	The secondary structure
		Temperature −1°C			Temperature 17°C		
C	*Arthrobacter* sp. 929901	−144.80	56.16	−136.90	−209.68	56.90	−200.52
	Sphingobium sp. 113573	−141.90	70.69	−135.20	−207.56	44.22	−197.71
	Streptomyces sp. 920852	−156.00	39.16	−155.90	−222.09	33.62	−210.79
	Nocardioides sp. 1033426	−155.50	61.70	−136.80	−226.07	37.11	−255.88
	Burkholderia sp.	−155.60	81.54	−133.20	−226.24	43.14	−217.22
	Achromobacter sp. 565246	−145.90	84.54	−125.80	−211.90	85.44	−167.67
	Rhodoplanes sp. 319907	−155.70	55.20	−143.20	−217.35	75.76	−183.44
DO	*Sphingomonas* sp. 243129	−137.10	83.30	−100.80	−200.58	43.93	−198.21
	Burkholderia sp. 281220	−151.80	96.36	−115.40	−222.88	48.23	−213.25
	Parvibaculum sp. 583617	−147.00	38.39	−145.40	−208.70	30.77	−206.91
	Luteibacter sp. 533999	−149.30	59.80	−142.40	−213.19	65.61	−168.76
	Salinibacterium sp. 1045797	−142.40	42.35	−138.60	−206.30	32.10	−203.98
	Rhodococcus sp. 278795	−155.00	73.53	−137.30	−225.04	45.64	−219.72
	Burkholderia sp.	−150.80	89.72	−126.40	−219.43	42.39	−204.18
	Methylibium sp.	−141.50	116.12	−99.40	−211.21	65.06	−194.84
	Nevskia sp.	−142.60	69.52	−137.20	−206.82	64.74	−186.13
	Sphingomonas sp.	−142.20	69.67	−127.60	−204.71	78.81	−164.16
	Gordonia sp. 558953	−151.70	71.09	−129.92	−218.43	78.50	−178.31
	Parvibaculum sp. 4336271	−147.30	83.64	−131.00	−209.36	81.36	−196.11
	Pseudoxanthomonas sp. 246060	−135.20	115.14	−102.90	−205.52	45.69	−193.57

	Pseudomonas sp. 239924	−143.90	73.51	−139.50	−210.56	57.35	−205.31
	Salinispora sp.	−163.20	57.67	−147.20	−229.98	34.26	−228.91
	Aquicella sp.	−141.20	70.03	−120.80	−205.51	44.37	−197.21
	Perlucidibaca sp. 848718	−141.50	81.85	−114.50	−205.34	54.45	−199.31
G	*Sphingomonas* sp. 1108960	−135.70	84.40	−106.70	−199.55	44.77	−198.02
	Bacillus sp.	−138.00	105.05	−91.50	−202.34	74.10	−190.02
G, DO	*Sphingomonas* sp.	−144.00	76.19	−125.20	−207.51	79.90	−169.75
	Achromobacter sp. 558264	−143.60	91.65	−121.40	−209.08	75.34	−179.00
	Sphingobium sp. 592615	−141.40	93.21	−110.20	−206.59	51.24	−191.16
	Mycobacterium sp. 1062748	−149.20	56.00	−143.00	−215.92	35.73	−213.63
G, C	*Pseudonocardia* sp. 2595164	−153.70	70.74	−134.10	−220.88	75.04	−175.65
	Ralstonia sp.	−147.90	95.78	−112.20	−214.68	63.66	−206.79
	Streptomyces sp. 1082846	−156.60	76.88	−127.00	−224.93	26.58	−220.44
	Bacillus sp. 1051517	−147.00	86.89	−129.80	−212.56	86.83	−193.64
G, DO, C	*Rhodanobacter* sp. 513602	−135.70	89.09	−106.70	−199.55	69.20	−198.02

C – bacteria identified in uncontaminated soil; DO – bacteria identified in soil contaminated with diesel fuel; G – bacteria identified in soil contaminated with gasoline; G, DO – bacteria common to soil contaminated with gasoline and diesel fuel; G, C – bacteria common to gasoline-contaminated and uncontaminated soil; G, DO, C – bacteria common to gasoline-contaminated, diesel-contaminated, and uncontaminated soil.

Table S3. Predicted protein properties calculated based on nucleotide sequences of fungus clones identified in soil from individual treatments. The calculations were performed using the ProtParam database program (https://web.expasy.org/protparam/) on the ExPasy portal.

Treatment	Fungal clones	Amino acid structure %				Molecular weight (kDa)	Instability index	Aliphatic index	GRAVY
		Ala	Cys	Gly	Thr				
C	*Mucor* sp. New.Reference	33.30	16.80	19.30	30.50	23.66	41.7	33.3	0.7
	Podila sp. New.Reference	30.80	21.10	15.40	32.60	19.42	**52.4**	**30.8**	0.8
	Linnemannia sp. New.Reference	31.10	21.10	15.60	31.20	18.55	46.5	31.1	0.8
	Minimedusa sp. New.Reference	29.10	20.00	19.60	31.30	23.15	57.0	29.1	0.7
	Humicola sp. SH1615601.08	23.20	27.50	24.00	25.30	19.62	61.3	23.2	0.8
	Fusarium sp. SH1212089.08	30.00	28.20	20.50	21.40	18.42	62.5	30.0	1.0
	Cladorrhinum sp. SH1505060.08	26.70	25.80	25.00	22.50	19.84	63.9	26.7	0.9
	Parachaetomium sp. New.Reference	23.90	30.80	24.40	20.90	19.63	55.8	23.9	1.0
DO	*Daedalea* sp. SH1571967.08	23.80	25.70	24.50	26.00	22.53	61.4	23.8	0.8
	Penicillium sp. New.Reference	19.80	33.20	28.30	18.60	20.60	49.6	19.8	0.9
G	*Talaromyces* clone SH1676308.08	20.10	32.10	28.50	19.30	20.73	55.1	20.1	0.9
	Penicillium sp. New.Reference	20.60	30.80	26.70	21.90	20.71	48.8	20.7	0.9
	Actinomucor sp. SH1621089.08	28.70	20.10	23.10	28.10	26.81	49.2	28.7	0.7
G, DO	*Penicillium* sp. New.Reference	20.90	30.70	27.00	21.30	20.40	50.3	30.9	0.9
G, C	*Pseudaleuria* sp. New.Reference	31.10	20.10	19.00	29.70	22.89	45.1	31.1	0.8
	Penicillium sp. SH1532997.08	20.30	32.50	27.60	19.50	20.56	47.8	20.3	0.9
	Mortierella sp. New.Reference	30.30	20.70	18.30	30.70	21.19	47.6	30.3	0.8
	Gymnostellatospora sp. New.Reference	23.40	26.60	25.00	25.00	20.75	61.0	23.4	0.8
	Parachaetomium sp. New.Reference	23.70	31.90	23.70	20.70	19.55	56.8	23.7	1.0
	Chaetomium sp. New.Reference	23.20	30.10	25.20	21.50	20.60	59.2	23.2	0.9
G,DO, C	*Penicillium* sp. SH1167746.08	19.90	33.30	27.60	19.10	20.59	45.1	19.9	0.9
	Penicillium sp. New.Reference	20.70	32.50	27.60	19.10	20.53	46.9	20.7	0.9
	Penicillium sp. SH3231803.08	23.40	29.10	24.20	23.40	20.52	49.6	23.4	0.9

GRAVY – grand average of hydropathicity; C – bacteria identified in uncontaminated soil; DO – bacteria identified in soil contaminated with diesel fuel; G – bacteria identified in soil contaminated with gasoline; G, DO – bacteria common to soil contaminated with gasoline and diesel fuel; G, C – bacteria common to gasoline-contaminated and uncontaminated soil; G, DO, C – bacteria common to gasoline-contaminated, diesel-contaminated, and uncontaminated soil.

Table S4. Minimum free energy (MFE), diversity of pz complexes, and predictable secondary structure of RNA based on fungus nucleotide sequences in two temperature ranges calculated using the Turner model (2024). The calculations were performed using the RNAfold program (http://rna.tbi.univie.ac.at//cgi-bin/RNAWebSuite/RNAfold.cgi).

Treatment	Fungal clones	MFE	Diversity (pz)	The secondary structure	MFE	Diversity (pz)	The secondary structure
		Temperature −1°C			Temperature 17°C		
C	*Mucor* sp. New.Reference	−61.40	63.06	−54.60	−118.20	47.58	−103.10
	Podila sp. New.Reference	−48.10	34.38	−46.50	−90.08	30.15	−80.59
	Linnemannia sp. New.Reference	−44.50	29.60	−42.30	−88.64	29.71	−84.16
	Minimedusa sp. New.Reference	−55.70	53.31	−47.30	−110.24	40.89	−96.30
	Humicola sp. SH1615601.08	−73.00	46.74	−64.80	−121.51	53.26	−106.98
	Fusarium sp. SH1212089.08	−50.80	36.62	−44.40	−90.15	39.87	−69.30
	Cladorrhinum sp. SH1505060.08	−78.20	50.54	−64.60	−128.65	40.65	−115.46
	Parachaetomium sp. New.Reference	−73.30	36.48	−70.70	−123.22	26.98	−117.67
DO	*Daedalea* sp. SH1571967.08	−73.60	56.58	−68.60	−129.10	40.27	−127.03
	Penicillium sp. New.Reference	−95.00	44.52	−82.20	−149.37	51.25	−113.37
G	*Talaromyces* clone SH1676308.08	−92.80	34.29	−85.50	−148.28	31.63	−141.81
	Penicillium sp. New.Reference	−83.30	63.20	−77.50	−139.17	18.75	−139.17
	Actinomucor sp. SH1621089.08	−86.60	74.61	−77.20	−159.82	44.32	−143.03
G, DO	*Penicillium* sp. New.Reference	−88.30	59.64	−64.70	−142.45	46.01	−118.67
G, C	*Pseudaleuria* sp. New.Reference	−65.30	52.64	−53.70	−117.20	74.89	−84.60
	Penicillium sp. SH1532997.08	−86.30	59.25	−65.00	−139.37	44.82	−129.60
	Mortierella sp. New.Reference	−54.10	48.76	−46.30	−105.71	26.66	−101.54
	Gymnostellatospora sp. New.Reference	−74.30	70.98	−44.60	−129.34	13.30	−129.34
	Parachaetomium sp. New.Reference	−72.80	42.43	−62.40	−120.55	42.97	−108.47
	Chaetomium sp. New.Reference	−83.20	55.77	−66.10	−137.20	33.15	−135.13
G, DO, C	*Penicillium* sp. SH1167746.08	−90.20	54.73	−74.70	−142.72	51.13	−116.41
	Penicillium sp. New.Reference	−86.30	59.25	−65.00	−139.37	44.82	−129.60
	Penicillium sp. SH3231803.08	−78.90	43.50	−64.20	−130.60	38.63	−127.84

C – bacteria identified in uncontaminated soil; DO – bacteria identified in soil contaminated with diesel fuel; G – bacteria identified in soil contaminated with gasoline; G, DO – bacteria common to soil contaminated with gasoline and diesel fuel; G, C – bacteria common to gasoline-contaminated and uncontaminated soil; G, DO, C – bacteria common to gasoline-contaminated, diesel-contaminated, and uncontaminated soil.
